# AIM2 forms a complex with Pyrin and ZBP1 to drive PANoptosis and host defense

**DOI:** 10.1038/s41586-021-03875-8

**Published:** 2021-09-01

**Authors:** SangJoon Lee, Rajendra Karki, Yaqiu Wang, Lam Nhat Nguyen, Ravi C. Kalathur, Thirumala-Devi Kanneganti

**Affiliations:** 1 Department of Immunology, St. Jude Children’s Research Hospital, Memphis, TN 38105, USA; 2 Department of Structural Biology, St. Jude Children’s Research Hospital, Memphis, TN 38105, USA

**Keywords:** AIM2, Pyrin, ZBP1, inflammasome, innate immunity, host defense, pyroptosis, apoptosis, necroptosis, PANoptosis, PANoptosome, inflammatory cell death, herpes simplex virus, *Francisella novicida*, ASC, caspase-1, caspase-8, caspase-3, caspase-7, MLKL, RIPK3

## Abstract

Inflammasomes are important sentinels of innate immune defense, sensing pathogens and inducing cell death in infected cells^1^. There are several inflammasome sensors that each detect and respond to specific pathogen- and damage-associated molecular patterns (PAMPs and DAMPs)^1^. In contrast to this one PAMP/DAMP to one sensor specificity, during infection, live pathogens can induce the release of multiple PAMPs and DAMPs, which could contemporaneously engage multiple inflammasome sensors^2–5^. Here we discovered that AIM2 regulated the innate immune sensors Pyrin and ZBP1 to drive inflammatory signaling and inflammatory cell death, PANoptosis, and provide host protection during infections with herpes simplex virus 1 (HSV1) and *Francisella novicida*. We also observed that AIM2, Pyrin and ZBP1 were members of a large multi-protein complex along with ASC, caspase-1, caspase-8, RIPK3, RIPK1 and FADD that drove inflammatory cell death. Collectively, our findings define a previously unknown regulatory connection and molecular interaction among AIM2, Pyrin and ZBP1 that drives assembly of an AIM2-mediated multi-protein complex that involves multiple inflammasome sensors and cell death regulators. These results represent a new paradigm in understanding the functions of these molecules in innate immunity and inflammatory cell death, suggesting new therapeutic targets for AIM2-, ZBP1- and Pyrin-mediated diseases.

Inflammasomes are critical components of the innate immune response^1^. Following exposure to PAMPs and DAMPs, inflammasome sensors form a multi-protein complex that activates caspase-1, leading to cleavage of its downstream substrates, inflammatory signaling and inflammatory cell death. Individual inflammasome sensors detect and respond to specific PAMPs and DAMPs, but emerging evidence suggests that multiple inflammasome sensors can be activated in response to disease, particularly live pathogen infection, due to the presence of multiple PAMPs and DAMPs^2–5^.

The AIM2 inflammasome is known to sense dsDNA and plays essential roles in development, infectious diseases, inflammatory diseases and cancer^6–11^. However, several critical functions for AIM2 beyond its canonically described role in inflammasome formation and pyroptosis have been observed that cannot be explained by our current understanding of the AIM2 inflammasome^2^. Herpes simplex virus 1 (HSV1), a dsDNA virus that causes lifelong incurable, recurrent pathologies, and *Francisella*, a gram-negative bacteria that can cause rapid lethality upon infection, are two diverse pathogens that are known to activate the AIM2 inflammasome and cell death^6,10–13^. To investigate whether cells exposed to numerous PAMPs and DAMPs during these viral and bacterial infections can simultaneously engage multiple inflammasome sensors, we infected wild type (WT) bone marrow-derived macrophages (BMDMs) and BMDMs singly deficient in several major inflammasome sensors with HSV1 or *F. novicida*. Both HSV1 and *F. novicida* infections induced AIM2-dependent and NLRP3- and NLRC4-independent cleavage of caspase-1, release of the inflammasome-dependent cytokines IL-1β and IL-18 and cell death (Fig. 1a–o, Extended Data Fig. 1a–i). However, inflammasome activation and cell death were also partially reduced in *Mefv*^–/–^ BMDMs during HSV1 and *F. novicida* infections (Fig. 1p–t, Extended Data Fig. 1j–l), suggesting that these infections activate Pyrin to drive AIM2-mediated inflammatory signaling and cell death.

Because there was residual AIM2-mediated caspase cleavage observed in *Mefv*^–/–^ BMDMs after HSV1 and *F. novicida* infections (Fig. 1p, Extended Data Fig. 1j), we screened several innate immune sensors to determine their involvement. Loss of the sensor ZBP1, which regulates inflammatory cell death in infectious and sterile conditions^14–16^, resulted in reduced caspase-1 cleavage, release of IL-1β and IL-18 and cell death compared with WT BMDMs after HSV1 and *F. novicida* infections (Fig. 1u–y, Extended Data Fig. 1m–o), while loss of other sensors or adaptors had no effect (Extended Data Fig. 2), suggesting that ZBP1 is also involved in HSV1- and *F. novicida*-induced inflammatory signaling and cell death.

To determine whether Pyrin and ZBP1 acted cooperatively or redundantly to decrease caspase-1 activation and cell death, we next used colchicine to inhibit Pyrin activation^17,18^ in *Zbp1*^–/–^ BMDMs. We found that colchicine treatment in *Zbp1*^–/–^ BMDMs further reduced cell death, cleavage of caspase-1 and release of IL-1β and IL-18 during HSV1 and *F. novicida* infections to mirror the levels observed in *Aim2*^–/–^ BMDMs (Fig. 2a, b, Extended Data Fig. 3), suggesting that ZBP1 cooperates with Pyrin to drive the AIM2-dependent responses. We also generated *Mefv*^–/–^*Zbp1*^–/–^ mice and found that cell death, cleavage of caspase-1 and release of IL-1β and IL-18 during HSV1 and *F. novicida* infections were further reduced in *Mefv*^–/–^*Zbp1*^–/–^ BMDMs compared with *Mefv*^–/–^ or *Zbp1*^–/–^ BMDMs, mirroring the levels observed in *Aim2*^–/–^ BMDMs (Fig. 2c–j, Extended Data Fig. 4a, b). These results suggest that Pyrin and ZBP1 cooperate to induce AIM2-mediated inflammatory signaling and cell death during both viral infection with HSV1 and bacterial infection with *F. novicida*. Similarly, we also observed activation of cell death and caspase-1 in human THP-1 macrophages during HSV1 infection, and this activation was inhibited by siRNA knockdown of *AIM2* or the combined knockdown of *ZBP1* and *MEFV* (Extended Data Fig. 4c–e), suggesting that the AIM2- and Pyrin and ZBP1-mediated cell death also occurs in human cells, the natural host of HSV1. Furthermore, cell death and caspase-1 cleavage were comparable between WT and *Mefv*^–/–^, *Zbp1*^–/–^ or *Mefv*^–/–^*Zbp1*^–/–^ BMDMs treated with the synthetic AIM2 inflammasome ligand poly(dA:dT) (Extended Data Fig. 4f–h), suggesting that infection is a unique trigger causing Pyrin and ZBP1 to cooperate to facilitate AIM2-mediated caspase-1 activation, cytokine release and cell death.

To understand how Pyrin was activated during HSV1 and *F. novicida* infections, we first evaluated RhoA-GTP activity, which is known to be inhibited to activate Pyrin in response to bacterial toxins, such as the *Clostridium difficile* toxin TcdB^17–21^. We found that TcdB-mediated inhibition of RhoA-GTP activity occurred in an AIM2- and ZBP1-independent manner (Extended Data Fig. 5a), while HSV1-mediated inhibition was AIM2-dependent and ZBP1-independent (Extended Data Fig. 5a). We next examined RhoA-GTP levels in *Aim2*^*–/–*^, *Asc*^*–/–*^ and *Casp1*^*–/–*^ BMDMs during HSV1 and *F. novicida* infections. While RhoA-GTP was absent in WT BMDMs upon infection, it was present in *Aim2*^*–/–*^, *Asc*^*–/–*^ and *Casp1*^*–/–*^ BMDMs (Extended Data Fig. 5b, c). In contrast, treatment with the synthetic AIM2 inflammasome ligand poly(dA:dT) did not inhibit RhoA-GTP activity in any of the genotypes tested (Extended Data Fig. 5d, e), suggesting that infection has a distinct ability to integrate the AIM2-mediated signaling and RhoA signaling cascades to influence Pyrin activation.

We then investigated how ZBP1 was activated during HSV1 and *F. novicida* infections. In the context of influenza A virus (IAV) infection, ZBP1 Zα domains sense nucleic acids to drive RIPK3-, caspase-8- and NLRP3-mediated inflammatory cell death^14,15,22^. Similarly, we found that HSV1- and *F. novicida*-induced cell death was reduced in *Zbp1*^ΔZa2/ΔZa2^ BMDMs compared to WT BMDMs and was comparable to that of *Zbp1*^*–/–*^ BMDMs (Extended Data Fig. 5f–i).

Our data show that HSV1 and *F. novicida* can activate multiple inflammasome sensors. Many pathogens can also induce multiple programmed cell death pathways, including pyroptosis, apoptosis and necroptosis^14,23^. Recent studies have found extensive crosstalk between programmed cell death pathways^14,24–27^, establishing the concept of PANoptosis^14,28–31^. Although inflammasome activation is primarily associated with GSDMD-mediated pyroptosis, the contribution of inflammasome components in driving PANoptosis is emerging. Therefore, we biochemically characterized the cell death induced by HSV1 and *F. novicida* infections. In WT cells, HSV1 and *F. novicida* activated key molecules involved in pyroptotic, apoptotic and necroptotic pathways (Fig. 3). However, we observed reduced activation of the pyroptotic molecules caspase-1, GSDMD and GSDME (Fig. 3a), apoptotic molecules caspase-8, −3 and −7 (Fig. 3b) and necroptotic molecules RIPK3 and MLKL (Fig. 3c) in *Mefv*^–/–^ and *Zbp1*^–/–^ BMDMs compared with WT BMDMs following HSV1 or *F. novicida* infection and a complete loss of activation in *Aim2*^–/–^ and *Mefv*^–/–^*Zbp1*^–/–^ BMDMs. Collectively, these data suggest that HSV1 and *F. novicida* infections induce inflammatory cell death, PANoptosis, dependent on AIM2 and the coordinated activation of Pyrin and ZBP1.

We next sought to understand the regulatory relationship between AIM2, Pyrin and ZBP1 during infection with HSV1 or *F. novicida.* Loss of AIM2 completely abrogated the inflammatory cell death, while loss of Pyrin or ZBP1 resulted in a partial reduction, suggesting that AIM2 functions upstream. We found that protein expression of Pyrin and ZBP1 was reduced in *Aim2*^–/–^ BMDMs after HSV1 or *F. novicida* infection (Fig. 4a, b). Additionally, their expression was also reduced in *Asc*^–/–^, *Casp1*^–/–^ and enzymatically inactive *Casp1*^*C284A/C284A*^ BMDMs as well as in WT BMDMs treated with the caspase-1 inhibitor VX-765 (Fig. 4c, d), but their expression was not reduced in *Gsdmd*^–/–^, *Casp8*^DA/DA^, *Casp7*^–/–^, *Casp3*^–/–^ or *Casp6*^–/–^ BMDMs after HSV1 and *F. novicida* infections (Extended Data Fig. 6a–c). Furthermore, expression of Pyrin and ZBP1 were not affected during IAV infection in *Aim2*^–/–^, *Asc*^–/–^, *Casp1*^–/–^ and *Nlrp3*^–/–^ BMDMs (Extended Data Fig. 6d). Therefore, AIM2-mediated signaling functions as an upstream regulator for the expression of Pyrin and ZBP1 in response to HSV1 and *F. novicida* infections.

To investigate the molecular mechanism underlying how AIM2, ASC and caspase-1 activity-dependent signals promote the expression of Pyrin and ZBP1 in infected cells, we assessed transcription and observed that HSV1- or *F. novicida*-infected *Aim2*^*–/–*^, *Asc*^*–/–*^ and *Casp1*^*–/–*^ BMDMs had reduced transcription of *Zbp1* and *Mefv* compared with the levels in WT cells (Extended Data Fig. 7a–d). Previously, we have shown that expression of Pyrin and ZBP1 can be induced by IFN signaling^14,32^. Therefore, we investigated whether AIM2 could induce IFN production to drive the expression of Pyrin and ZBP1. We found that WT BMDMs produced IFN-β in response to HSV1 (MOI, 10; 6 h post-infection) or *F. novicida* infection (MOI, 1; 3 h post-infection), while *Aim2*^*–/–*^, *Asc*^*–/–*^ and *Casp1*^*–/–*^ BMDMs did not (Extended Data Fig. 7e, f). Additionally, the expression of Pyrin and ZBP1 was restored upon complementation of IFN-β in the AIM2-, ASC- and caspase-1-deficient cells (Extended Data Fig. 7g, h). The differences in IFN production observed here are in contrast to previous studies with *F. novicida*, where these knockouts have not been reported to have a profound loss of type I IFN production. These differences might be due to differing timepoints and MOIs: *Asc*^*–/–*^ BMDMs had slightly reduced IFN-β release 5 h post-infection (MOI, 100)^33^ and comparable *Ifnb* mRNA levels 9 h post-infection (MOI, 100)^34^; *Aim2*^*–/–*^ BMDMs had significantly increased IFN-β release 6 h post-infection (MOI, 250)^11^; and *Asc*^*–/–*^ and *Casp1/11*^*–/–*^ BMDMs had significantly increased IFN-β release 6 h post-infection (MOI, 50)^35^. These differences suggest that type I IFN release in response to *F. novicida* may be timepoint- and/or MOI-dependent.

Next, we sought to understand the molecular relationship between AIM2, Pyrin and ZBP1 in inducing inflammatory cell death, PANoptosis, during HSV1 and *F. novicida* infections. We hypothesized that these molecules would all be part of the molecular scaffold that allows contemporaneous engagement of key molecules from pyroptosis, apoptosis and necroptosis^28,29,31,36^. We observed interactions of ASC with AIM2, Pyrin, ZBP1, caspase-1, caspase-8, RIPK3, RIPK1 and FADD by immunoprecipitation following HSV1 and *F. novicida* infections (Fig. 4e), and these interactions were not observed in *Aim2*^*–/–*^, *Mefv*^*–/–*^*Zbp1*^*–/–*^, *Ripk3*^*–/–*^*Casp8*^*–/–*^ or *Ripk3*^*–/–*^*Fadd*^*–/–*^ BMDMs (Extended Data Fig. 8). In contrast, treatment with poly(dA:dT) allowed interactions between endogenous ASC, AIM2 and caspase-1, but not Pyrin, ZBP1, caspase-8, RIPK3, RIPK1 or FADD (Fig. 4e), suggesting that infection has a distinct ability to form this AIM2 multi-protein cell death-inducing complex. We have termed this complex the AIM2 PANoptosome. We also observed that ASC specks colocalized with AIM2, Pyrin and ZBP1 collectively in the same cell at 12 hours post-infection with HSV1 or *F. novicida* (Fig. 4f, g, Extended Data Fig. 9a, b). Similarly, ASC specks also colocalized with caspase-8 and RIPK3 in the same cell (Extended Data Fig. 9c–f). Furthermore, the formation of this complex was required for cell death, as its disruption through the deletion of key components inhibited cell death in response to HSV1 and *F. novicida* infections (Figure 2c, d, Extended Data Fig. 9g, h).

We then extended our findings to *in vivo* models of HSV1 and *F. novicida* infection. Lung lysates from infected WT mice showed activation of key pyroptotic, apoptotic and necroptotic molecules and increased expression of Pyrin and ZBP1, but this activation and increased expression was not found in *Aim2*^–/–^ mice (Extended Data Fig. 10a, b). The number of plaque forming units (PFU) and colony forming units (CFU) for HSV1 and *F. novicida*, respectively, was increased in *Aim2*^*–/–*^*, Mefv*^*–/–*^*, Zbp1*^*–/–*^ *and Mefv*^*–/–*^*Zbp1*^*–/–*^ BMDMs and in the tissue of *Aim2*^*–/–*^ mice (Fig. 4h, Extended Data Fig. 10c–e). Additionally, all mice lacking AIM2 succumbed to HSV1 infection within 8 days and mice lacking Pyrin or ZBP1 succumbed within 13 days, while ~70% of the WT mice survived (Fig. 4i). Similarly, all mice lacking AIM2 succumbed to *F. novicida* infection within 4 days and mice lacking Pyrin or ZBP1 succumbed within 9 or 11 days, respectively, whereas ~80% of the WT mice survived (Extended Data Fig. 10f). These results highlight an important role for the AIM2-regulated expression of Pyrin and ZBP1 and inflammatory cell death, PANoptosis, in host defense against HSV1 and *F. novicida* infections.

In summary, we discovered that AIM2, Pyrin and ZBP1 form a multi-protein complex termed the AIM2 PANoptosome via interactions with ASC to induce PANoptosis during HSV1 and *F. novicida* infections. Loss of AIM2 reduced the expression of Pyrin and ZBP1 during these infections, indicating that AIM2-mediated signaling functions as an upstream regulator of Pyrin and ZBP1 to control assembly and activation of the AIM2 PANoptosome. The ability of HSV1 and *F. novicida* to activate AIM2 while also engaging the ZBP1 Zα domain and inhibiting Rho-GTP activity gives these pathogens the unique ability to form this AIM2-, Pyrin- and ZBP1-containing complex. Other pathogens that can engage multiple sensors may also form similar complexes with shared central components.

Furthermore, we observed new roles for ZBP1 in innate immunity. ZBP1 is known to sense influenza viral Z-RNA to promote binding of RIPK3, caspase-6 and the NLRP3 inflammasome to form the ZBP1 PANoptosome complex and drive PANoptosis^14,15,22,31^. The multi-protein complex identified here is mediated by AIM2 but may be similar to this ZBP1 PANoptosome, as it shares the same cell death effectors, including caspase-8 and RIPK3, and also leads to the induction of PANoptosis, characterized by activation of pyroptotic, apoptotic and necroptotic molecules. Additionally, we discovered that ZBP1 also interacts and localizes with ASC and acts as an inflammasome sensor to induce caspase-1 cleavage and inflammatory cell death in response to HSV1 and *F. novicida* infections. Furthermore, AIM2 regulated the expression of Pyrin and ZBP1 during HSV1 and *F. novicida* infections, but not during IAV infection.

Overall, our findings identify a critical interaction between AIM2, Pyrin and ZBP1 that drives innate immune responses during pathogen infection. This regulatory mechanism defines the molecular basis of how infection with live pathogens that liberate numerous PAMPs or DAMPs can trigger the coordinated activation of innate immune and cell death signaling components to form a multi-protein complex and cause inflammatory cell death and cytokine release to shape the immune response and host defense.

## METHODS

### Mice

C57BL/6J [wide type (WT)], *Mefv*^*−/−*^ (ref. ^21^), *Aim2*^*−/−*^ (ref. ^33^), *Nlrp3*^*−/−*^ (ref. ^37^), *Nlrc4*^*−/−*^ (ref. ^38^), *Zbp1*^*−/−*^ (ref. ^39^), *Tlr3*^*−/−*^ (ref. ^40^), *Trif*^*−/−*^ (ref. ^41^), *Mda5*^*−/−*^ (ref. ^42^), *Mavs*^*−/−*^ (ref. ^43^), *Nlrp6*^*−/−*^ (ref. ^44^), *Nlrp12*^*−/−*^ (ref. ^45^), *Asc*^*−/−*^ (ref. ^46^), *Casp1*^*−/−*^ (ref. ^47^), *Casp1*^*C284A/C284A*^ (ref. ^48^), *Gsdmd*^*−/−*^ (ref. ^49^), *Casp8*^*DA/DA*^ (ref. ^50^), *Casp7*^–/–^ (ref. ^51^), *Casp3*^–/–^ (ref. ^52^), *Casp6*^*−/−*^ (Jackson Laboratory, 006236; ref. ^31^), *Ripk3*^–/–^ (ref. ^53^), *Ripk3*^–/–^*Casp8*^–/–^ (ref. ^54^) and *Ripk3*^–/–^*Fadd*^*–/–*^ (ref. ^55^) mice have been described previously. *Mefv*^*−/−*^ mice were crossed with *Zbp1*^*−/−*^ mice to generate *Mefv*^*−/−*^ *Zbp1*^*−/−*^ homozygous knockouts. All mice were bred and maintained in a specific pathogen-free facility at the Animal Resources Center at St. Jude Children’s Research Hospital and were backcrossed to the C57BL/6 background (J substrain) for at least 10 generations. Both male and female mice were used in this study; age- and sex-matched 6- to 8-week-old mice were used for *in vivo* and 6- to 12-week-old mice were used for *in vitro* studies. Cohoused animals were used for *in vivo* analyses. Mice were maintained in 20–23.3 degrees Celsius and 30–70% humidity with a 12 h light/dark cycle and were fed standard chow. Animal studies were conducted under protocols approved by the St. Jude Children’s Research Hospital committee on the Use and Care of Animals.

### Cell culture

Primary bone marrow-derived macrophages (BMDMs) were cultivated for 6 days in DMEM (Thermo Fisher Scientific, 12440–053) supplemented with 10% FBS (Biowest, S1620), 30% L929-conditioned media, 1% non-essential amino acids (Thermo Fisher Scientific, 11140–050) and 1% penicillin and streptomycin (Thermo Fisher Scientific, 15070–063). BMDMs were then seeded into 12-well plates at a density of 1 million cells per well and incubated overnight before use. THP-1 cells (ATCC® TIB-202™) were grown in RPMI 1640 with 10% FBS and differentiated into macrophages in RPMI 1640 medium containing 20% FBS and 100 ng/ml PMA for 2 days. The THP-1 cell line was purchased directly from ATCC and was not further authenticated in our lab. Cells were tested for mycoplasma contamination using mycoplasma detection PCR and were found to be negative for mycoplasma contamination.

### Virus and bacteria culture

Human herpes simplex virus 1 (HF strain) (ATCC; VR-260) was propagated in Vero cells, and the virus titer was measured by plaque assay in Vero cells. The influenza A virus (A/Puerto Rico/8/34, H1N1 [PR8]) was prepared as previously described^31^ and propagated from 11-day-old embryonated chicken eggs by allantoic inoculation. Influenza A virus titer was measured by plaque assay in MDCK cells. *Salmonella* Typhimurium strain SL1344 was inoculated into Luria-Bertani (LB) broth (MP Biomedicals, 3002–031) and incubated overnight under aerobic conditions at 37°C. *S.* Typhimurium was then sub-cultured (1:10) for 3 h at 37°C in fresh LB broth to generate bacteria grown to log phase. *Clostridium difficile* strain r20291 AB^-^ and AB^+^ strains (provided by Dr. N. Minton^56^) were streaked onto brain heart infusion agar (BD Biosciences, 211065) and incubated overnight at 37°C in an anaerobic chamber. Single colonies were inoculated into tryptone-yeast extract medium and grown overnight at 37°C anaerobically. *Francisella novicida* strain U112 was prepared as previously described^6^ and was grown in BBL™ Trypticase™ Soy Broth (TSB) (211768, BD) supplemented with 0.2% L-cysteine (Fisher) overnight under aerobic conditions at 37°C. Bacteria were subcultured (1:10) in fresh TSB supplemented with 0.2% L-cysteine for 3 h and resuspended in PBS. Gentamicin (50 μg/ml, Gibco) was added 3 h post-infection to kill extracellular *F. novicida*.

### Cell stimulation and infection

For ligand-mediated AIM2 inflammasome activation, 2 μg of poly(dA:dT) (InvivoGen, tlrl-patn) was resuspended in PBS and mixed with 0.6 μl of Xfect polymer in Xfect reaction buffer (Clontech Laboratories, Inc, 631318). After 10 min, DNA complexes were added to BMDMs in Opti-MEM (ThermoFisher Scientific, 31985–070), followed by incubation for 5 h. For NLRP3 inflammasome activation, BMDMs were primed for 4 h with 500 ng/mL ultrapure LPS from *E. coli* (0111:B4) (Invivogen, tlrl-3pelps) and then stimulated for 45 min with 20 μM nigericin (Sigma, N7143). For NLRC4 inflammasome activation, *S.* Typhimurium was infected at an MOI of 1 for 2 h. For Pyrin inflammasome activation, Toxin AB^+^ *C. difficile* (2 × 10^7^ CFU/ml) were spun down, and the supernatant was sterilized using 0.22 μM filters and then added to BMDMs for 12 h. For HSV1 (6, 12 or 16 h; MOI 10) and IAV (16 h; MOI 10) infections, cells were infected in DMEM plain media (Sigma, D6171). For *F. novicida* infection, cells were infected in DMEM (ThermoFisher Scientific, 11995–065) at a MOI of 50 (12 or 16 h) to determine caspase, GSDMD, and GSDME cleavage and for cell death analyses, at a MOI of 150 (24 h) to determine phosphorylated MLKL and RIPK3 levels or at a MOI of 1 (3 h) for IFN analyses. For inhibition of the Pyrin inflammasome, 30 μM colchicine (Sigma, C9754) was added to BMDMs 1 h before infection. For inhibition of caspase-1, 20 μM VX-765 (Chemietek, CT-VX765) was added to BMDMs 1 h before the infection. For the IFN-β treatment, 50 ng/mL of IFN-β (PBL Assay, 12400–1) was added to BMDMs 3 h after infections. To measure *in vitro* viral titer, the indicated BMDMs were infected with HSV1 (MOI 10) for 12 h, and the supernatants were used for plaque assays in Vero cells. To measure *in vitro F. novicida* levels, cells were infected at a MOI of 100, and cells were harvested with PBS containing 0.5% Triton X-100 at 6 or 12 h post-infection. The *F. novicida* were then streaked onto TSB plates supplemented with 0.2% L-cysteine and grown overnight at 37°C anaerobically.

### siRNA-mediated gene silencing

The Accell human siRNA SMARTPools against *AIM2* (E-011951–00-0010), *MEFV* (E-011081–00-0010) and *ZBP1* (E-014650–00-0010) genes were purchased (Horizon). THP-1 macrophages were transfected with siRNA using Accell siRNA delivery media according to the manufacturer’s instructions (Horizon). As a negative control, non-targeting control siRNA (D-001910–01-50) was used.

### Real time qRT-PCR analysis

RNA was extracted using TRIzol according to the manufacturer’s instructions (Life Technologies). Isolated RNA was reverse transcribed into cDNA using the First-Strand cDNA Synthesis Kit (Life Technologies). Real-time qPCR was performed on an ABI 7500 real-time PCR instrument with 2× SYBR Green (Applied Biosystems). Primer sequences used in this study were as follows: 5`-AAGAGTCCCCTGCGATTATTTG-3` and 5`-TCTGGATGGCGTTTGAATTGG-3` for *Zbp1*; 5`-TCATCTGCTAAACACCCTGGA-3` and 5`-GGGATCTTAGAGTGGCCCTTC-3` for *Mefv*; 5`-CGTCCCGTAGACAAAATGGT-3` and 5`-TTGATGGCAACAATCTCCAC-3` for *Gapdh*.

### Measurement of active RhoA-GTP levels

BMDMs (2 × 10^6^ cells) were seeded in 6 well plates and transfected with 0.5 μg/ml TcdB, 0.5 μg poly(dA:dT) or infected with HSV (MOI 10) or *F. novicida* (MOI 100). RhoA-GTP levels were measured using the RhoA G-LISA Activation Assay kit (BK121, Cytoskeleton) and normalized to total RhoA levels, which were measured using the Total RhoA ELISA kit (BK150, Cytoskeleton) according to the manufacturer’s instructions. For pull-down assays, a GST-tagged Rho binding domain (RBD) in the Rho effector protein was used, which has been shown to bind specifically to the GTP-bound form of RhoA^17,19^. RhoA-GTP in BMDMs (2 × 10^6^ cells) was pull-down with Rhotekin-RBD beads using the RhoA activation Assay Biochem Kit (BK036, Cytoskeleton) according to the manufacturer’s instructions.

### *In vivo* infection

Age- and sex-matched, 6- to 8-week-old WT and *Aim2*^*–/*^,^*–*^ *Mefv*^*–/–*^ and *Zbp1*^*–/–*^ mice were used for infections. For HSV1 infection, mice were anesthetized with 250 mg/kg Avertin and then infected intranasally with HSV1 in 50 μl PBS containing around 5 × 10^7^ PFU. Infected mice were monitored over a period of 14 days for survival. For the *F. novicida* infection, *F. novicida* strain U112 was grown in TSB supplemented with 0.2% L-cysteine overnight at 37°C and then 1:10 subcultured for 4 h before infection. Mice were infected subcutaneously with 5 × 10^5^ CFU of *F. novicida* in 200 μl PBS. Infected mice were monitored over a period of 12 days for survival. Lungs, livers and/or spleens harvested at the indicated time points were homogenized in 1 ml PBS for viral titers to be enumerated by plaque assays or for bacterial titers to be streaked onto TSB supplemented with 0.2% L-cysteine plates and grown overnight at 37°C anaerobically.

### Immunoblot analysis

Immunoblotting was performed as described previously^57^. Briefly, for caspase analysis, BMDMs were lysed along with the supernatant using 50 μl caspase lysis buffer (1 × protease inhibitors, 1 × phosphatase inhibitors, 10% NP-40 and 25 mM DTT) followed by the addition of 100 μl 4 × SDS loading buffer. For signaling analysis, the BMDM supernatants were removed at the indicated time points, and cells were washed once with PBS, after which cells were lysed with RIPA buffer. Proteins from lung, spleen and liver tissues were extracted using RIPA buffer supplemented with protease and phosphatase inhibitors (Roche), and 30 μg per sample was loaded on the gel. Proteins were separated by electrophoresis through 8%–12% polyacrylamide gels. Following electrophoretic transfer of proteins onto PVDF membranes (Millipore, IPVH00010), non-specific binding was blocked by incubation with 5% skim milk; then membranes were incubated with the following primary antibodies: anti-caspase-1 (AdipoGen, AG-20B-0042, 1:1000), anti-caspase-3 (CST, #9662, 1:1000), anti-cleaved caspase-3 (CST, #9661, 1:1000), anti-caspase-7 (CST, #9492, 1:1000), anti-cleaved caspase-7 (CST, #9491, 1:1000), anti-caspase-8 (CST, #4927, 1:1000), anti-cleaved caspase-8 (CST, #8592, 1:1000), anti-pRIPK3 (CST, #91702S, 1:1000), anti-RIPK3 (ProSci, #2283, 1:1000), anti-pMLKL (CST, #37333, 1:1000), anti-MLKL (Abgent, AP14272b, 1:1000), anti-GSDMD (Abcam, ab209845, 1:1000), anti-GSDME (Abcam, ab215191, 1:1000), anti-Pyrin (Abcam, ab195975, 1:1000), anti-AIM2 (Abcam, ab119791, 1:1000), anti-ZBP1 (AdipoGen, AG-20B-0010, 1:1000), anti-ASC (Millipore, #04–147, 1:1000 or AdipoGen, AG-25B-006-C100, 1:1000), anti-RIPK1 (CST, 3493, 1:1000), anti-FADD (Millipore, 05–486, 1:1000), anti-β-actin (Proteintech, 66009–1-IG, 1:5000), human anti-caspase-1 (R&D systems; Cat# MAB6215, 1:1000), human anti-β-actin (CST, 4970, 1:1000). Membranes were then washed and incubated with the appropriate horseradish peroxidase (HRP)-conjugated secondary antibodies (1:5000 dilution; Jackson Immuno Research Laboratories, anti-rabbit [111–035-047], anti-mouse [315–035-047]) for 1 h. Proteins were visualized by using Luminata Forte Western HRP Substrate (Millipore, WBLUF0500), and membranes were developed with an Amersham imager; images were analyzed with ImageJ (v1.53a).

### Real-time cell death analysis

Real-time cell death assays were performed using an IncuCyte S3 imaging system (Essen Biosciences). BMDMs were seeded in 12-well plates (10^6^ cells/well) and stimulated. After infection, 100 nM SYTOX Green (Thermo Fisher Scientific, S7020) was added. The images were acquired every 1 h at 37°C and 5% CO_2_. The resulting images were analyzed using the software package supplied with the IncuCyte imager, which counts the number of Sytox Green-positive BMDM nuclei (Sytox^+^ BMDM nuclei) present in each image.

### Immunoprecipitation

Immunoprecipitation was performed as described previously^58^. Briefly, after HSV1 or *F. novicida* infection or poly(dA:dT) treatment, WT BMDMs were lysed in a buffer containing 20 mM Tris-HCl (pH 7.4), 100 mM NaCl, 30 mM KCl and 0.1% NP-40. After centrifugation at 16,000 g for 10 min, the lysates were incubated with either IgG control antibody (CST, 3900S) or anti-ASC antibody (AdipoGen; AG-25B-006-C100) with protein A/G PLUS-Agarose (Santa Cruz Biotechnology) overnight at 4°C. After washing with the above buffer, the immunoprecipitated proteins were harvested by boiling in 1 × SDS loading buffer at 100°C for 5 min.

### Immunofluorescence staining

Immunofluorescence staining was performed as described previously^59^. Briefly, after infection, BMDMs were fixed in 4% paraformaldehyde (ChemCruz, sc-281692) for 10 min and permeabilized with PBS containing 0.5% Triton X-100 for 3 min. Cells were then incubated in PBS containing 1% skim milk for 1 h. Alexa Fluor 488 (ThermoFisher, A20181)-conjugated anti-ASC (Millipore, 04–147), Alexa Fluor 532 (ThermoFisher, A20182)-conjugated anti-AIM2 (Abcam, ab119791), Alexa Fluor 568 (ThermoFisher, A20184)-conjugated anti-Pyrin (Abcam, ab195975), Alexa Fluor 647 (ThermoFisher, A20186)-conjugated anti-ZBP1 (AdipoGen, AG-20B-0010), Alexa Fluor 568 (ThermoFisher, A20184)-conjugated anti-RIPK3 (ProSci, #2283) or Alexa Fluor 647 (ThermoFisher, A20186)-conjugated anti-caspase-8 (Enzo, 1G12) were made according to the manufacturer’s instructions, and the coverslips were incubated with indicated antibodies (1:100) for 1 h. Cells were counterstained with DAPI mounting medium (Invitrogen, P36931). Images were acquired by confocal laser scanning microscopy (LSM780; Carl Zeiss) using × 63 Apochromat objective.

### Cytokine analysis

Cytokines were detected by using multiplex ELISA (Millipore, MCYTOMAG-70K), IL-18 ELISA (Invitrogen, BMS618–3) or IFN-β ELISA (BioLegend, 439408) according to the manufacturer’s instructions.

### Statistical analysis

GraphPad Prism 8.0 software was used for data analysis. Data are presented as mean ± SEM. Statistical significance was determined by t tests (two-tailed) for two groups or one-way ANOVA with Dunnett’s multiple comparisons test for more groups. Survival analysis was performed using the log-rank (Mantel-Cox) test. *P* values less than 0.05 were considered statistically significant where *P < 0.05, **P < 0.01, ***P < 0.001 and ****P < 0.0001.

## Extended Data

**Extended Data Fig. 1.**
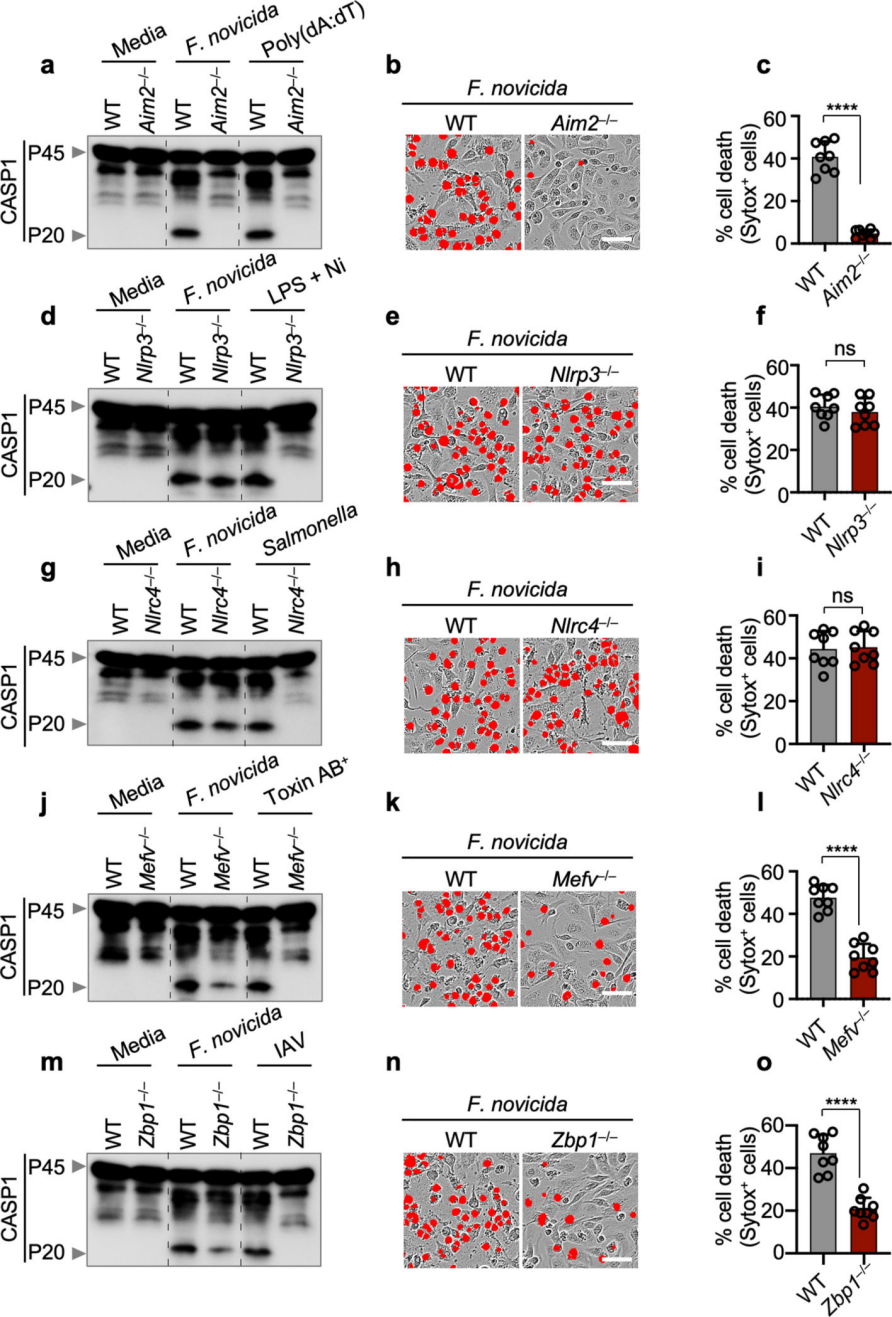
*F. novicida* induces AIM2-, Pyrin-, ZBP1-mediated caspase-1 activation, cytokine release and cell death. **a,** Immunoblot analysis of pro–caspase-1 (CASP1; P45) and cleaved CASP1 (P20) in *F. novicida*-infected or poly(dA:dT)-transfected wild type (WT) or *Aim2*^*−/−*^ bone marrow-derived macrophages (BMDMs). **b,** Cell death in BMDMs after *F. novicida* infection for 16 h. Red indicates dead cells. **c**, Quantification of the cell death in (**b**). **d–f,** Immunoblot analysis of CASP1 (**d**), cell death images at 16 h post-infection (**e**), and cell death quantification (**f**) from WT or *Nlrp3*^*−/−*^ BMDMs after *F. novicida* infection or LPS plus nigericin (LPS + Ni) treatment. **g–i,** Immunoblot analysis of CASP1 (**g**), cell death images at 16 h post-infection (**h**), and cell death quantification (**i**) from WT or *Nlrc4*^*−/−*^ BMDMs after *F. novicida* or *Salmonella* Typhimurium infection. **j–l,** Immunoblot analysis of CASP1 (**j**), cell death images at 16 h post-infection (**k**), and cell death quantification (**l**) from WT or *Mefv*^*−/−*^ BMDMs after *F. novicida* infection or *C. difficile* Toxin AB^+^ supernatant treatment. **m–o,** Immunoblot analysis of CASP1 (**m**), cell death images at 16 h post-infection (**n**), and cell death quantification (**o**) from WT or *Zbp1*^*−/−*^ BMDMs after *F. novicida* or influenza A virus (IAV) infection. **a, d, g, j, m,** Data are representative of at least three independent experiments. **b, e, h, k, n,** Images are representative of at least three independent experiments. Scale bar, 50 μm. **c, f, i, l, o,** Data are mean ± s.e.m. ns, not significant; ****P < 0.0001 (two-tailed t-test; *n* = 8 from 4 biologically independent samples). Exact *P* values are presented in Supplementary Table 1. For gel source data, see Supplementary Figure 1.

**Extended Data Fig. 2.**
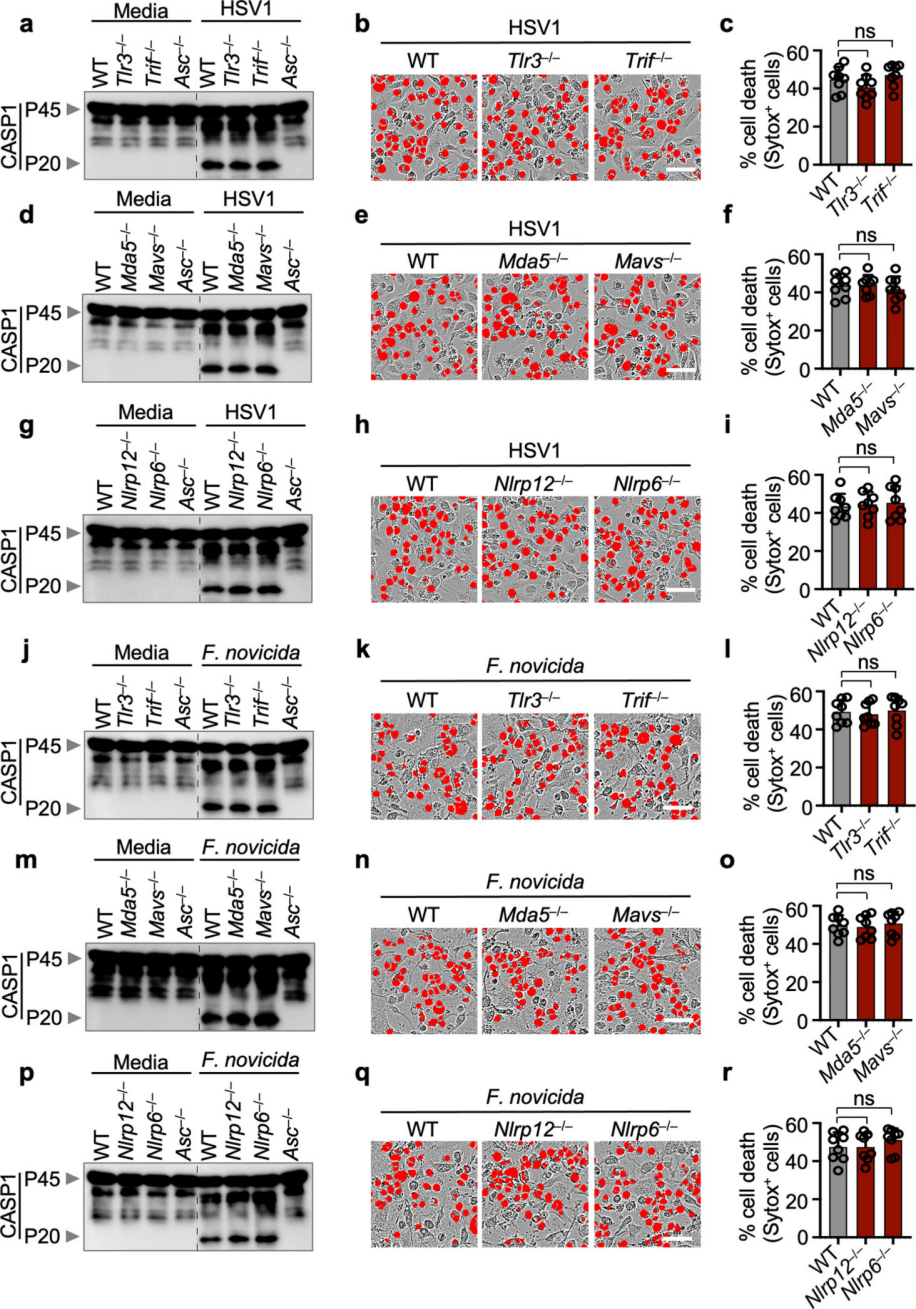
Innate immune sensors TLR3, MDA5, NLRP6 and NLRP12 and adaptors Trif and MAVS are not required for caspase-1 activation and cell death after HSV1 and *F. novicida* infections. **a,** Immunoblot analysis of pro–caspase-1 (CASP1; P45) and cleaved CASP1 (P20) in HSV1-infected wild type (WT), *Tlr3*^*−/−*^*, Trif*^*−/−*^ or *Asc*^*−/−*^ bone marrow-derived macrophages (BMDMs). **b,** Cell death in BMDMs after HSV1 infection for 16 h. Red indicates dead cells. **c**, Quantification of the cell death in (**b**). **d–f,** Immunoblot analysis of CASP1 (**d**), cell death images at 16 h post-infection (**e**), and cell death quantification (**f**) from WT, *Mda5*^*−/−*^ or *Mavs*^*−/−*^ BMDMs after HSV1 infection. **g–i,** Immunoblot analysis of CASP1 (**g**), cell death images at 16 h post-infection (**h**), and cell death quantification (**i**) from WT, *Nlrp6*^*−/−*^ or *Nlrp12*^*−/−*^ BMDMs after HSV1 infection. **j–l,** Immunoblot analysis of CASP1 (**j**), cell death images at 16 h post-infection (**k**), and cell death quantification (**l**) from WT, *Tlr3*^*−/−*^ or *Trif*^*−/−*^ BMDMs after *F. novicida* infection. **m–o,** Immunoblot analysis of CASP1 (**m**), cell death images (**n**), and cell death quantification (**o**) from WT, *Mda5*^*−/−*^ or *Mavs*^*−/−*^ BMDMs after *F. novicida* infection. **p–r,** Immunoblot analysis of CASP1 (**p**), cell death images at 16 h post-infection (**q**), and cell death quantification (**r**) from WT, *Nlrp6*^*−/−*^ or *Nlrp12*^*−/−*^ BMDMs after *F. novicida* infection. **a, d, g, j, m, p,** Data are representative of at least three independent experiments. **b, e, h, k, n, q,** Images are representative of at least three independent experiments. Scale bar, 50 μm. **c, f, i, l, o, r,** Data are mean ± s.e.m. ns, not significant (one-way ANOVA with Dunnett’s multiple comparisons test; *n* = 8 from 4 biologically independent samples). Exact *P* values are presented in in Supplementary Table 1. For gel source data, see Supplementary Figure 1.

**Extended Data Fig. 3.**
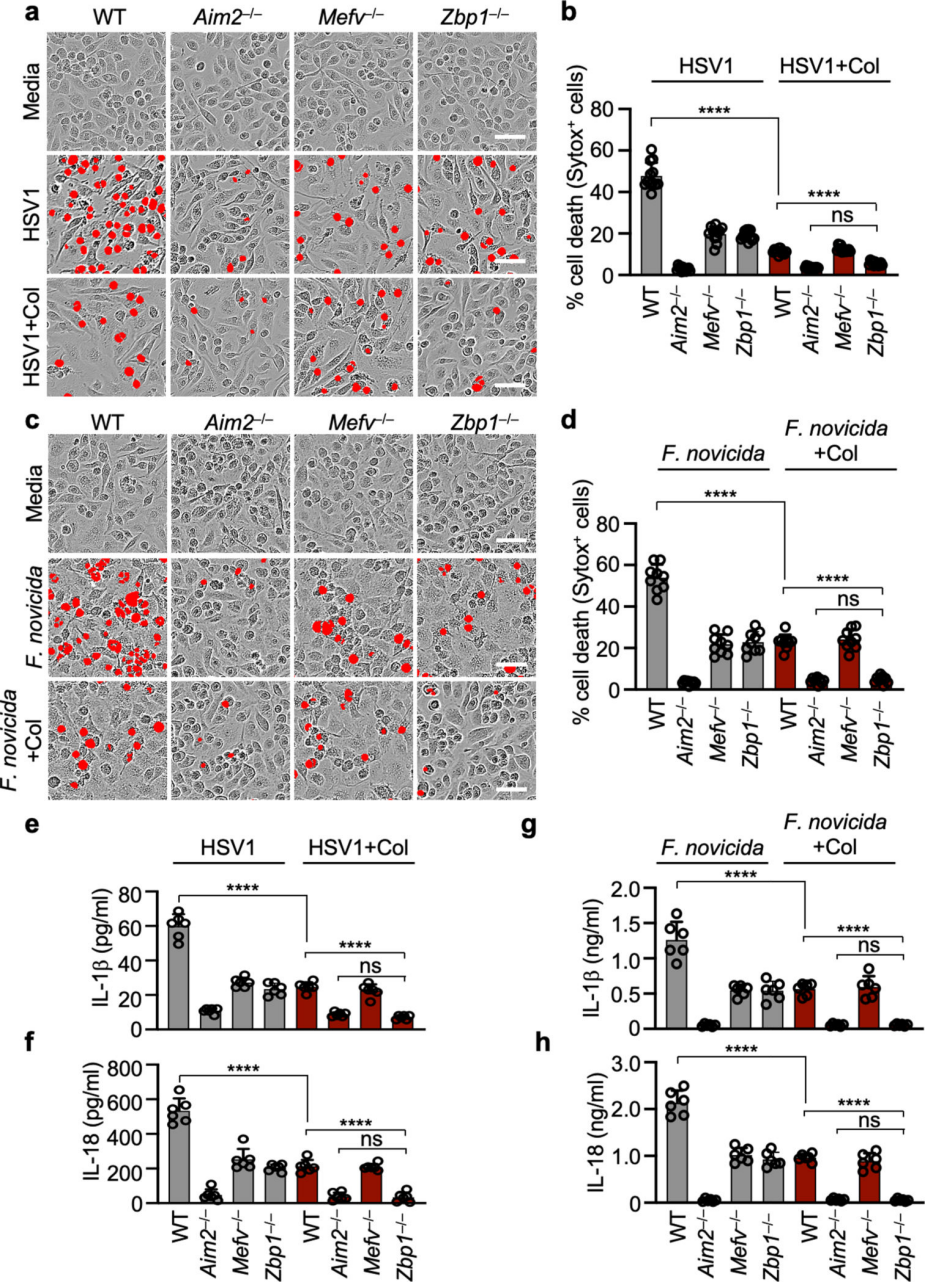
ZBP1 cooperates with Pyrin to drive AIM2-mediated cell death and cytokine release. **a,** Cell death in bone marrow-derived macrophages (BMDMs) after HSV1 infection with or without colchicine (Col). Red indicates dead cells. Data are representative of at least three independent experiments. Scale bar, 50 μm. **b,** Quantification of the cell death from (a). Data are mean ± s.e.m. ns, not significant; ****P < 0.0001 (one-way ANOVA with Dunnett’s multiple comparisons test; *n* = 12 from 3 biologically independent samples). **c,** Cell death in BMDMs after *F. novicida* infection with or without Col. Red indicates dead cells. Data are representative of at least three independent experiments. Scale bar, 50 μm. **d,** Quantification of the cell death from (c). Data are mean ± s.e.m. ns, not significant; ****P < 0.0001 (one-way ANOVA with Dunnett’s multiple comparisons test; *n* = 9 from 3 biologically independent samples). **e–h,** Release of IL-1β (**e, g**) or IL-18 (**f, h**) following HSV1 (**e, f**) or *F. novicida* (**g, h**) infections with or without Col. Data are mean ± s.e.m. ns, not significant; ****P < 0.0001 (one-way ANOVA with Dunnett’s multiple comparisons test; *n* = 6 from 3 biologically independent samples). Exact *P* values are presented in Supplementary Table 1.

**Extended Data Fig. 4.**
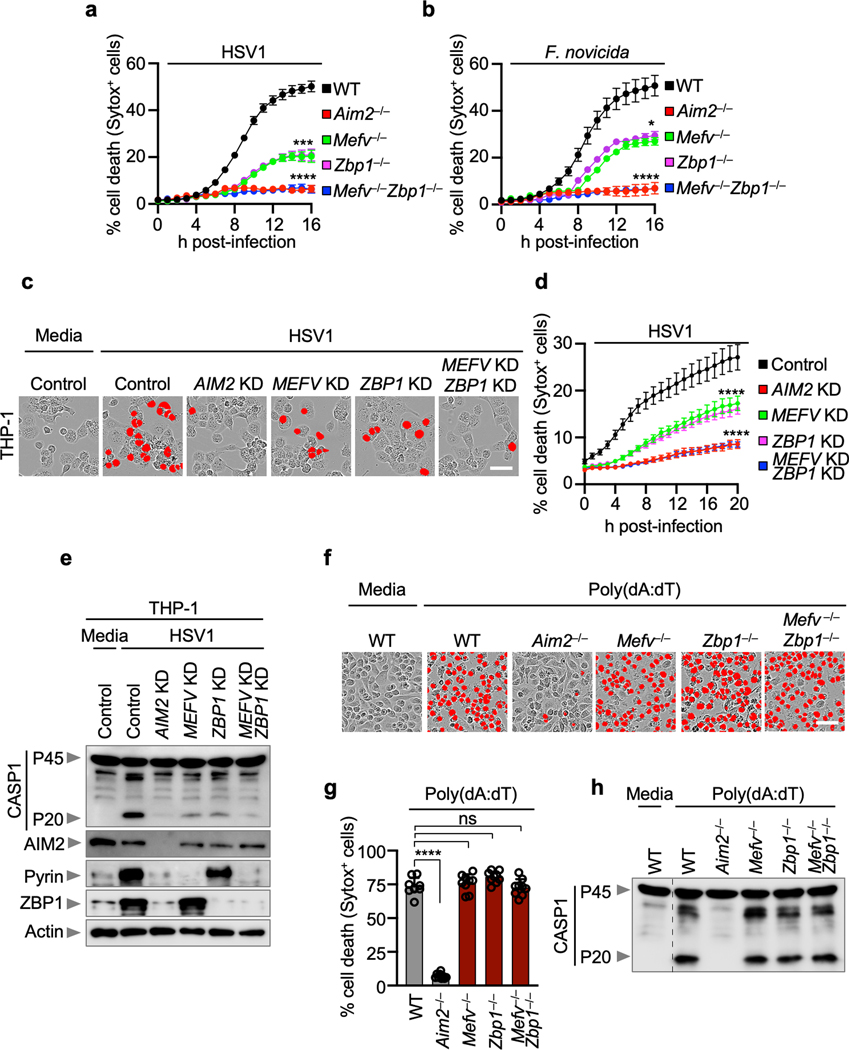
Pyrin and ZBP1 are required for AIM2-mediated cell death following HSV1 infection, but not in response to poly(dA:dT). **a, b,** Quantification of cell death in wild type (WT), *Aim2*^*−/−*^*, Mefv*^*−/−*^, *Zbp1*^*−/−*^ or *Mefv*^*−/−*^*Zbp1*^*−/−*^ bone marrow-derived macrophages (BMDMs) over time during HSV1 (**a**) and *F. novicida* (**b**) infections. Data are mean ± s.e.m. *P < 0.05; ***P < 0.001; ****P < 0.0001 (one-way ANOVA with Dunnett’s multiple comparisons test; *n* = 4). Data are representative of at least three independent experiments. **c,** Cell death in THP-1 macrophages treated with control siRNA (Control) or siRNA targeted to *AIM2* (*AIM2* KD), *MEFV* (*MEFV* KD) and/or *ZBP1* (*ZBP1* KD) after HSV1 infection. Red indicates dead cells. Images are representative of at least three independent experiments. Scale bar, 50 μm. **d,** Quantification of the cell death from (c). Data are mean ± s.e.m. ****P < 0.0001 (one-way ANOVA with Dunnett’s multiple comparisons test; *n* = 4). Data are representative of at least three independent experiments. **e,** Immunoblot analysis of caspase-1 (CASP1) activation and AIM2, Pyrin and ZBP1 expression in the indicated THP-1 cells. Data are representative of two independent experiments. **f,** Cell death in WT, *Aim2*^*−/−*^*, Mefv*^*−/−*^, *Zbp1*^*−/−*^ or *Mefv*^*−/−*^*Zbp1*^*−/−*^ BMDMs after poly(dA:dT) transfection. Red indicates dead cells. Images are representative of at least three independent experiments. Scale bar, 50 μm. **g,** Quantification of the cell death from (f). Data are mean ± s.e.m. ns, not significant; ****P < 0.0001 (one-way ANOVA with Dunnett’s multiple comparisons test; *n* = 9 from 3 biologically independent samples). Exact *P* values are presented in Supplementary Table 1. **h,** Immunoblot analysis of CASP1 in the indicated BMDMs after poly(dA:dT) transfection. Data are representative of at least three independent experiments. For gel source data, see Supplementary Figure 1.

**Extended Data Fig. 5.**
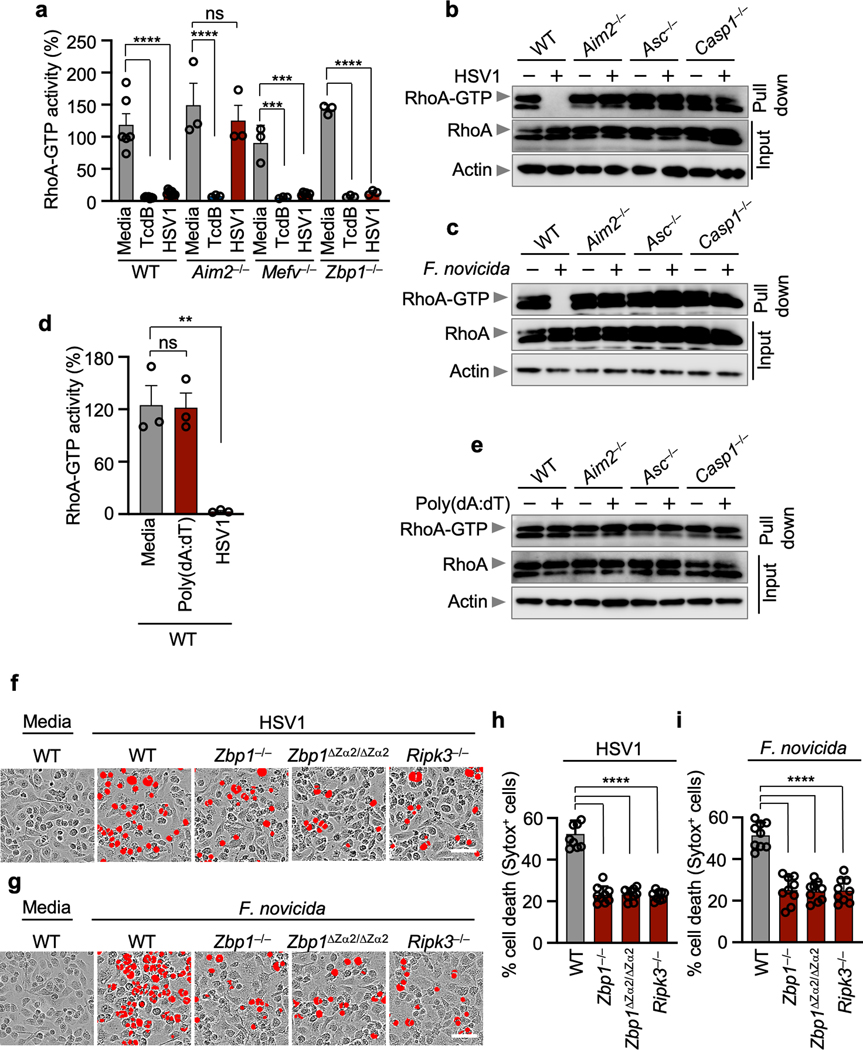
AIM2 acts as an upstream regulator of RhoA modifications, and the ZBP1 Zα2 domain is required for cell death. **a,** RhoA-GTP activity in wild type (WT), *Aim2*^*−/−*^, *Mefv*^*−/−*^ or *Zbp1*^*−/−*^ bone marrow-derived macrophages (BMDMs) infected with HSV1 or treated with TcdB for 12 h. Data are mean ± s.e.m. from three independent experiments. ns, not significant; ***P < 0.001; ****P < 0.0001 (one-way ANOVA with Dunnett’s multiple comparisons test; *n* = 3, 6, 7 or 9). **b, c,** Activated RhoA (RhoA-GTP) assessed using a pull-down assay with Rhotekin-RBD beads from WT, *Aim2*^*−/−*^*, Asc*^*−/−*^ or *Casp1*^*−/−*^ BMDMs infected with HSV1 (**b**) or *F. novicida* (**c**). Data are representative of at least three independent experiments. **d,** RhoA-GTP activity in WT BMDMs infected with HSV1 or transfected with poly(dA:dT) for 12 h. Data are mean ± s.e.m. from three independent experiments. ns, not significant; **P < 0.01 (one-way ANOVA with Dunnett’s multiple comparisons test; *n* = 3). **e,** Activated RhoA (RhoA-GTP) assessed using a pull-down assay with Rhotekin-RBD beads from WT, *Aim2*^*−/−*^*, Asc*^*−/−*^ or *Casp1*^*−/−*^ BMDMs transfected with poly(dA:dT). Data are representative of at least three independent experiments. **f, g** Cell death in WT, *Zbp1*^*−/−*^*, Zbp1*^*ΔZa2/ΔZa*2^ or *Ripk3*^*−/−*^ BMDMs after HSV1 (**f**) or *F. novicida* (**g**) infections. Red indicates dead cells. Images are representative of at least three independent experiments. Scale bar, 50 μm. **h, i,** Quantification of the cell death from panel f (**h**) or panel g (**i**). Data are mean ± s.e.m. ****P < 0.0001 (one-way ANOVA with Dunnett’s multiple comparisons test; *n* = 9 from 3 biologically independent samples). Exact *P* values are presented in Supplementary Table 1. For gel source data, see Supplementary Figure 1.

**Extended Data Fig. 6.**
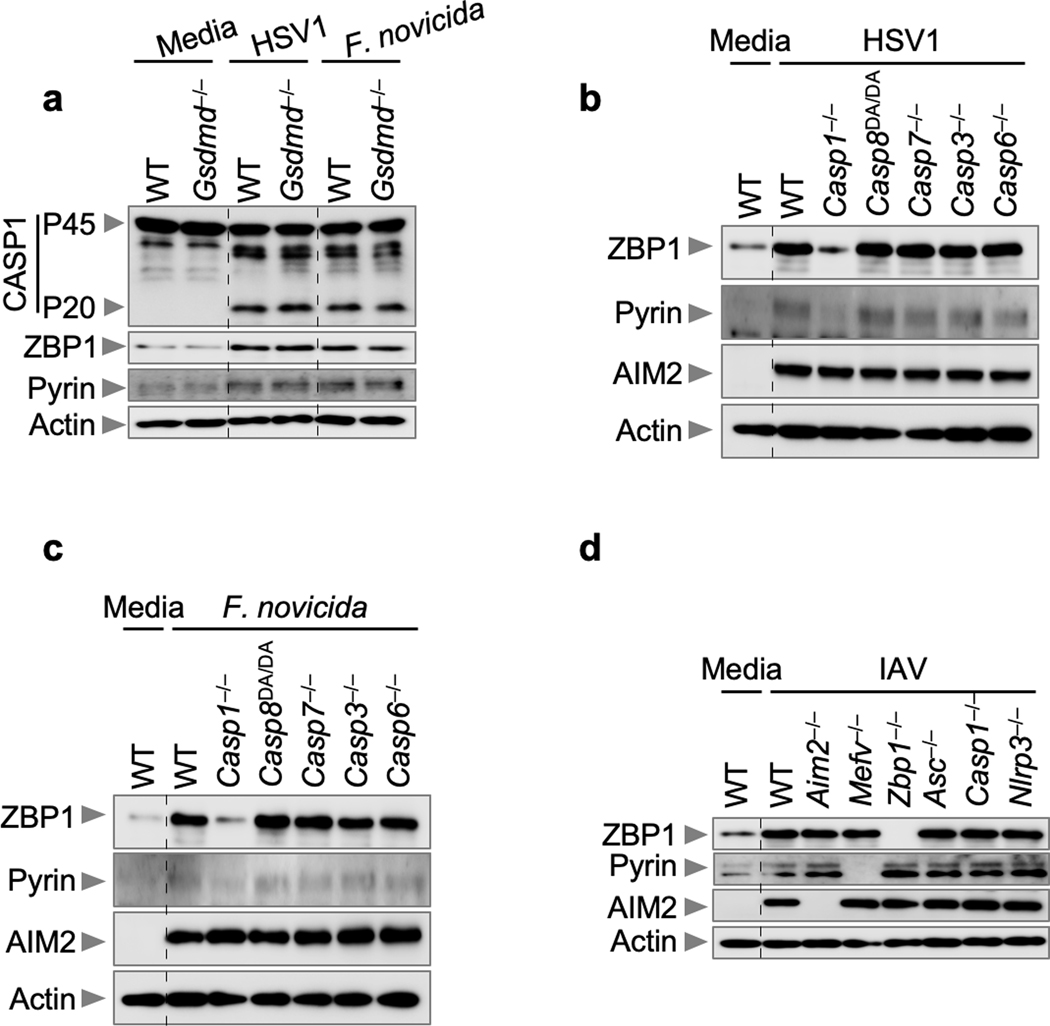
The expression of Pyrin and ZBP1 is not regulated by GSDMD or caspase-8, −7, −3 or −6 during HSV1 or *F. novicida* infections. **a,** Immunoblot analysis of caspase-1 (CASP1) activation and Pyrin and ZBP1 expression in wild type (WT) or *Gsdmd*^*−/−*^ bone marrow-derived macrophages (BMDMs) after HSV1 or *F. novicida* infection. Data are representative of at least three independent experiments. **b, c,** Immunoblot analysis of ZBP1, Pyrin and AIM2 expression in the indicated BMDMs after HSV1 (**b**) or *F. novicida* (**c**) infections. Data are representative of at least three independent experiments. **d,** Immunoblot analysis of ZBP1, Pyrin and AIM2 expression in the indicated BMDMs after influenza A virus (IAV) infection. Data are representative of at least three independent experiments. For gel source data, see Supplementary Figure 1.

**Extended Data Fig. 7.**
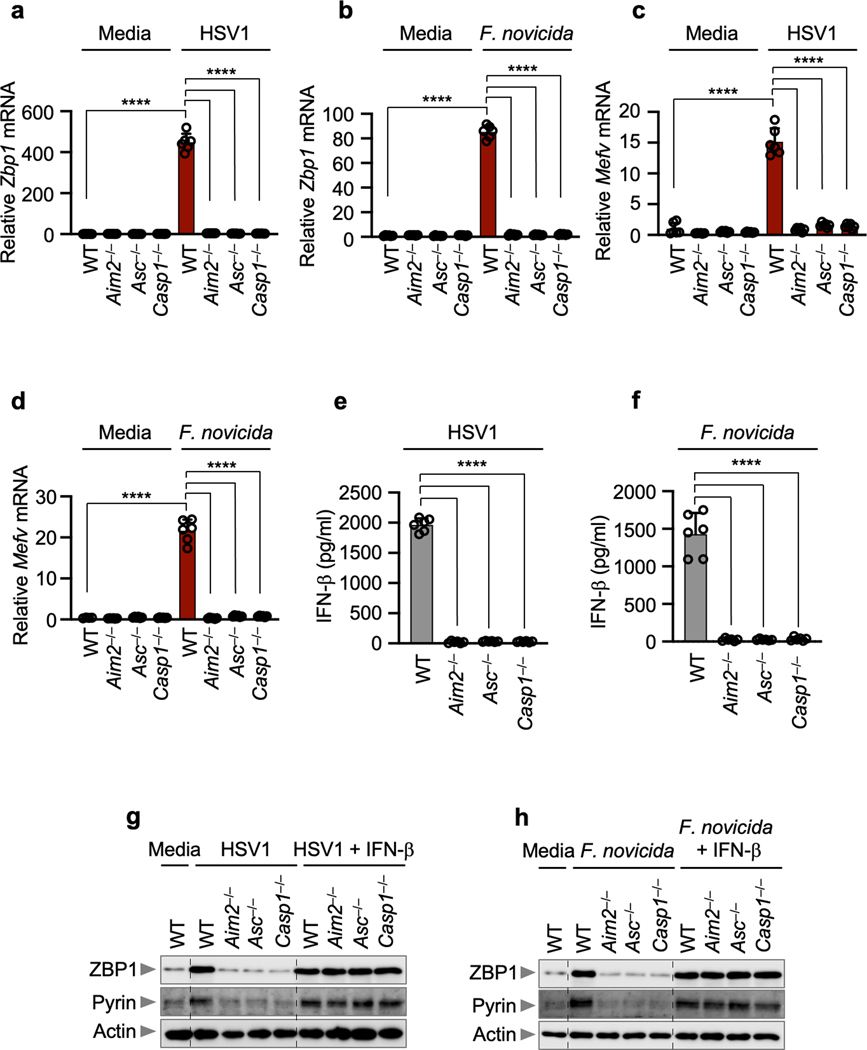
The expression of Pyrin and ZBP1 is regulated by AIM2 during HSV1 and *F. novicida* infections. **a–d,** Relative expression of *Zbp1* (**a, b**) and *Mefv* (**c, d**) in wild type (WT), *Aim2*^*−/−*^*, Asc*^*−/−*^ or *Casp1*^*−/−*^ bone marrow-derived macrophages (BMDMs) after HSV1 (**a, c**) or *F. novicida* (**b, d**) infections. Expression presented relative to that of the control gene *Gapdh*. Data are mean ± s.e.m. from three independent experiments. ****P < 0.0001 (one-way ANOVA with Dunnett’s multiple comparisons test, *n* = 6). **e, f,** Release of IFN-β following HSV1 (**e**) or *F. novicida* (**f**) infections. Data are mean ± s.e.m. ****P < 0.0001 (one-way ANOVA with Dunnett’s multiple comparisons test; *n* = 6 from 3 biologically independent samples). Exact *P* values are presented in Supplementary Table 1. **g, h,** Immunoblot analysis of ZBP1, Pyrin and AIM2 expression in the indicated BMDMs after HSV1 (**g**) or *F. novicida* (**h**) infections with or without IFN-β treatment. Data are representative of at least three independent experiments. For gel source data, see Supplementary Figure 1.

**Extended Data Fig. 8.**
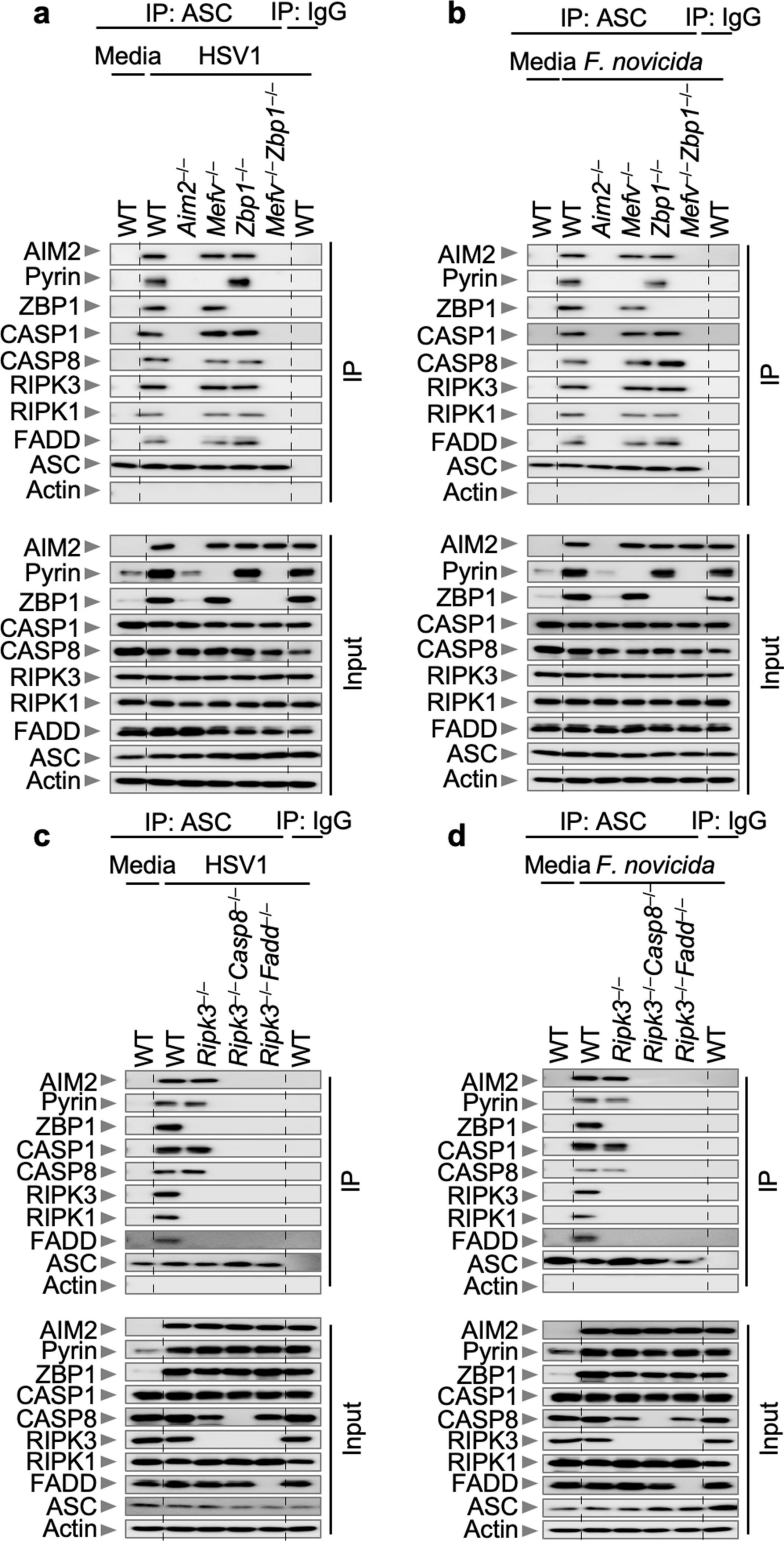
Loss of AIM2 or combined loss of Pyrin and ZBP1 prevents the formation of the AIM2 complex during HSV1 and *F. novicida* infections. **a, b,** Immunoprecipitation (IP) in wild type (WT), *Aim2*^*−/−*^*, Mefv*^*−/−*^*, Zbp1*^*−/−*^ or *Mefv*^*−/−*^*Zbp1*^*−/−*^ bone marrow-derived macrophages (BMDMs) with either IgG control antibodies or anti-ASC antibodies after HSV1 (**a**) or *F. novicida* (**b**) infection. Data are representative of three independent experiments. **c, d,** IP in WT, *Ripk3*^*−/−*^*, Ripk3*^*−/−*^*Casp8*^*−/−*^ or *Ripk3*^*−/−*^*Fadd*^*−/−*^ BMDMs with either IgG control antibodies or anti-ASC antibodies after HSV1 (**c**) or *F. novicida* (**d**) infection. Data are representative of three independent experiments. For gel source data, see Supplementary Figure 1.

**Extended Data Fig. 9.**
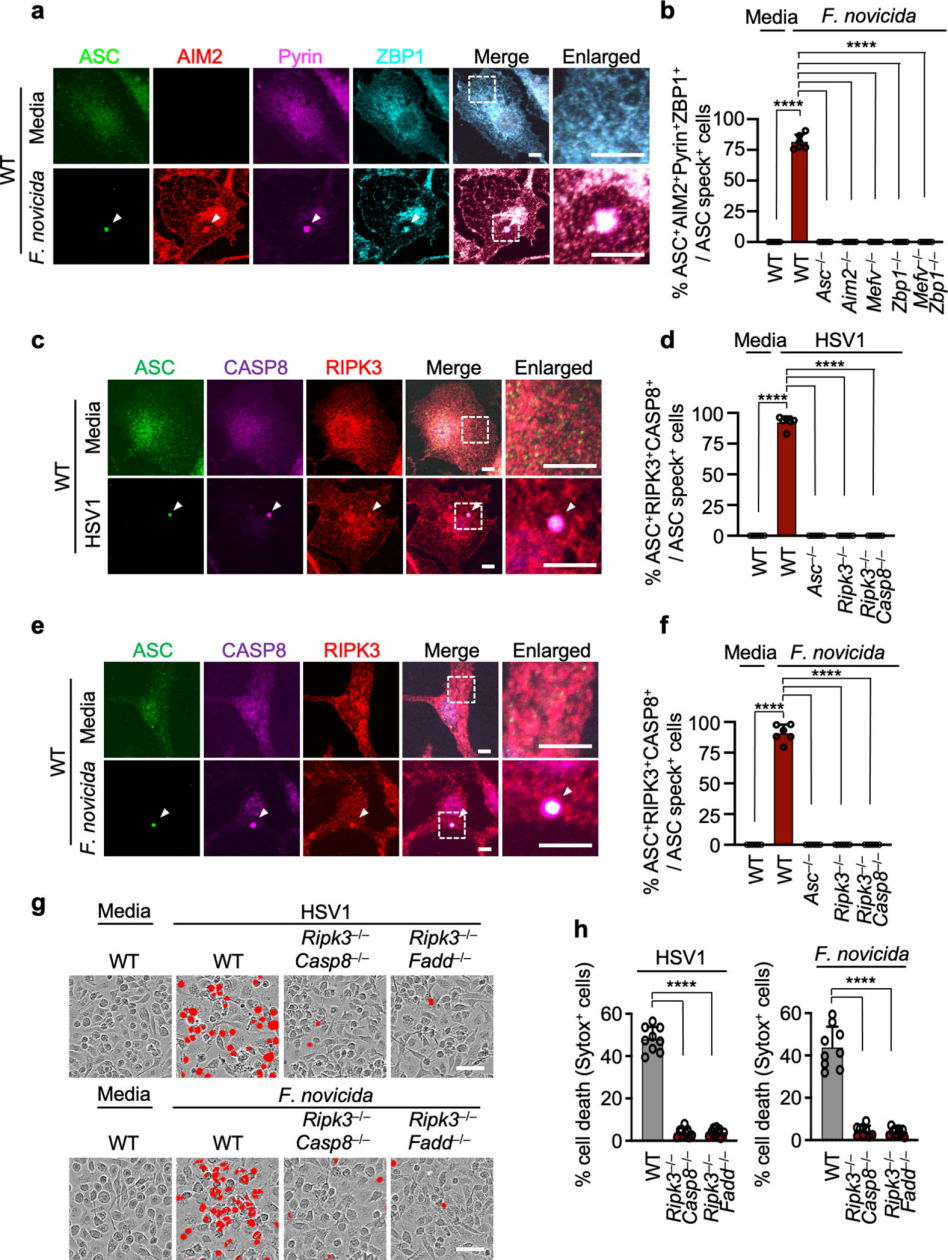
ASC speck colocalizes with AIM2, Pyrin and ZBP1, caspase-8 and RIPK3 in the same cell during HSV1 and *F. novicida* infections, and formation of this complex drives cell death. **a,** Immunofluorescence images of wild type (WT) bone marrow-derived macrophages (BMDMs) at 12 h after *F. novicida* infection. Scale bars, 5 μm. Arrowheads indicate the ASC speck. Images are representative of three independent experiments. **b,** Quantification of the percentage of cells with ASC^+^AIM2^+^Pyrin^+^ZBP1^+^ specks among the ASC speck^+^ cells. Data are mean ± s.e.m. ****P < 0.0001 (one-way ANOVA with Dunnett’s multiple comparisons test; *n* = 6 from 3 biologically independent samples). **c,** Immunofluorescence images of WT BMDMs at 12 h after HSV1 infection. Scale bars, 5 μm. Arrowheads indicate the ASC speck. Images are representative of three independent experiments. **d,** Quantification of the percentage of cells with ASC^+^RIPK3^+^CASP8^+^ specks among the ASC speck^+^ cells. Data are mean ± s.e.m. ****P < 0.0001 (one-way ANOVA with Dunnett’s multiple comparisons test; *n* = 6 from 3 biologically independent samples). **e,** Immunofluorescence images of WT BMDMs at 12 h after *F. novicida* infection. Scale bars, 5 μm. Arrowheads indicate the ASC speck. Images are representative of three independent experiments. **f,** Quantification of the percentage of cells with ASC^+^RIPK3^+^CASP8^+^ specks among the ASC speck^+^ cells. Data are mean ± s.e.m. ****P < 0.0001 (one-way ANOVA with Dunnett’s multiple comparisons test; *n* = 6 from 3 biologically independent samples). **g,** Cell death in WT, *Ripk3*^*−/−*^*Casp8*^*−/−*^ or *Ripk3*^*−/−*^*Fadd*^*−/−*^ BMDMs at 16 h post-infection with HSV1 or *F. novicida*. Red indicates dead cells. Data are representative of at least three independent experiments. Scale bar, 50 μm. **h,** Quantification of the cell death from (g). Data are mean ± s.e.m. ****P < 0.0001 (one-way ANOVA with Dunnett’s multiple comparisons test; *n* = 9 from 3 biologically independent samples). Exact *P* values are presented in Supplementary Table 1.

**Extended Data Fig. 10.**
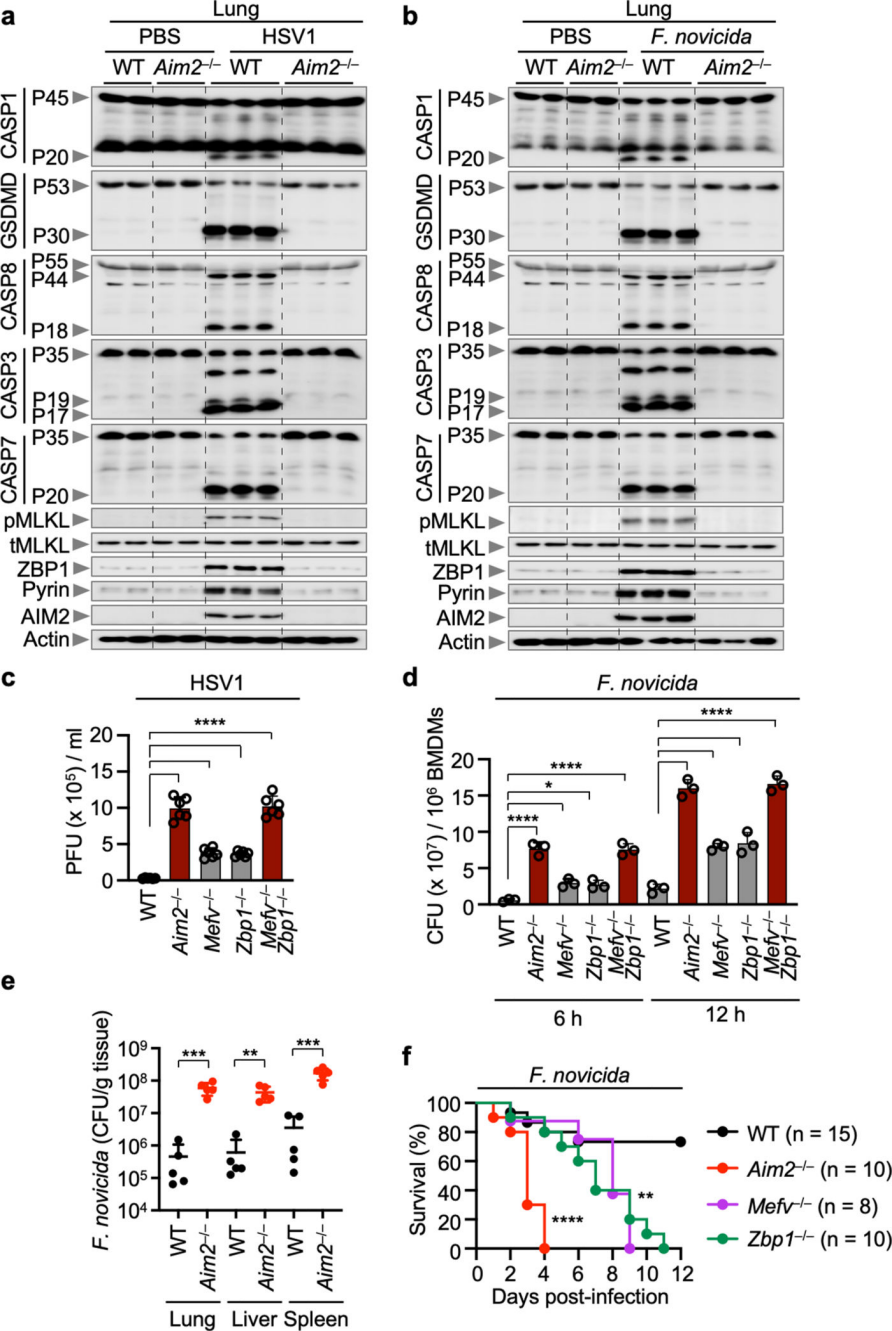
AIM2 regulates Pyrin and ZBP1 expression *in vivo*, and AIM2 provides host protection against HSV1 and *F. novicida.* **a, b,** Immunoblot analysis of pro- (P45) and activated (P20) caspase-1 (CASP1), pro- (P53) and activated (P30) gasdermin D (GSDMD), pro- (P55) and cleaved (P18) caspase-8 (CASP8), pro- (P35) and cleaved (P17/P19) caspase-3 (CASP3), pro- (P35) and cleaved (P20) caspase-7 (CASP7), phosphorylated MLKL (pMLKL), total MLKL (tMLKL), ZBP1, Pyrin and AIM2 in lung from uninfected animals (PBS) or wild type (WT) or *Aim2*^*−/−*^ mice 3 days after HSV1 (**a**) or *F. novicida* (**b**) infection. Each lane indicates independent biological replicates. **c,** Viral quantification in WT, *Aim2*^*−/−*^*, Mefv*^*−/−*^*, Zbp1*^*−/−*^ or *Mefv*^*−/−*^*Zbp1*^*−/−*^ BMDMs at 16 h post-infection with HSV1. Data are mean ± s.e.m. ****P < 0.0001 (one-way ANOVA with Dunnett’s multiple comparisons test; *n* = 6 from 3 biologically independent samples). **d,** Bacterial quantification in WT, *Aim2*^*−/−*^*, Mefv*^*−/−*^*, Zbp1*^*−/−*^ or *Mefv*^*−/−*^*Zbp1*^*−/−*^ BMDMs after *F. novicida* infection. Data are mean ± s.e.m. *P < 0.05 and ****P < 0.0001 (one-way ANOVA with Dunnett’s multiple comparisons test; *n* = 3 from 3 biologically independent samples). **e,** *In vivo* bacterial quantification in lung, liver or spleen from WT or *Aim2*^*−/−*^ mice 2 days after *F. novicida* infection (*n* = 5). Each symbol represents one mouse. Data are pooled from two independent experiments. Data are mean ± s.e.m. **P < 0.01 and ***P < 0.001 (two-tailed t-test). **f,** Survival of WT, *Aim2*^*−/−*^, *Mefv*^*−/−*^ and *Zbp1*^*−/−*^ mice infected subcutaneously with 5 × 10^5^ CFU of *F. novicida* in 200 μl PBS. Survival data are pooled from three independent experiments. **P < 0.01; ****P < 0.0001 (log-rank (Mantel-Cox) test). Exact *P* values are presented in Supplementary Table 1. For gel source data, see Supplementary Figure 1.

## Supplementary Material

Supplementary Figure 1

Supplementary Table 1

## Figures and Tables

**Fig. 1.**
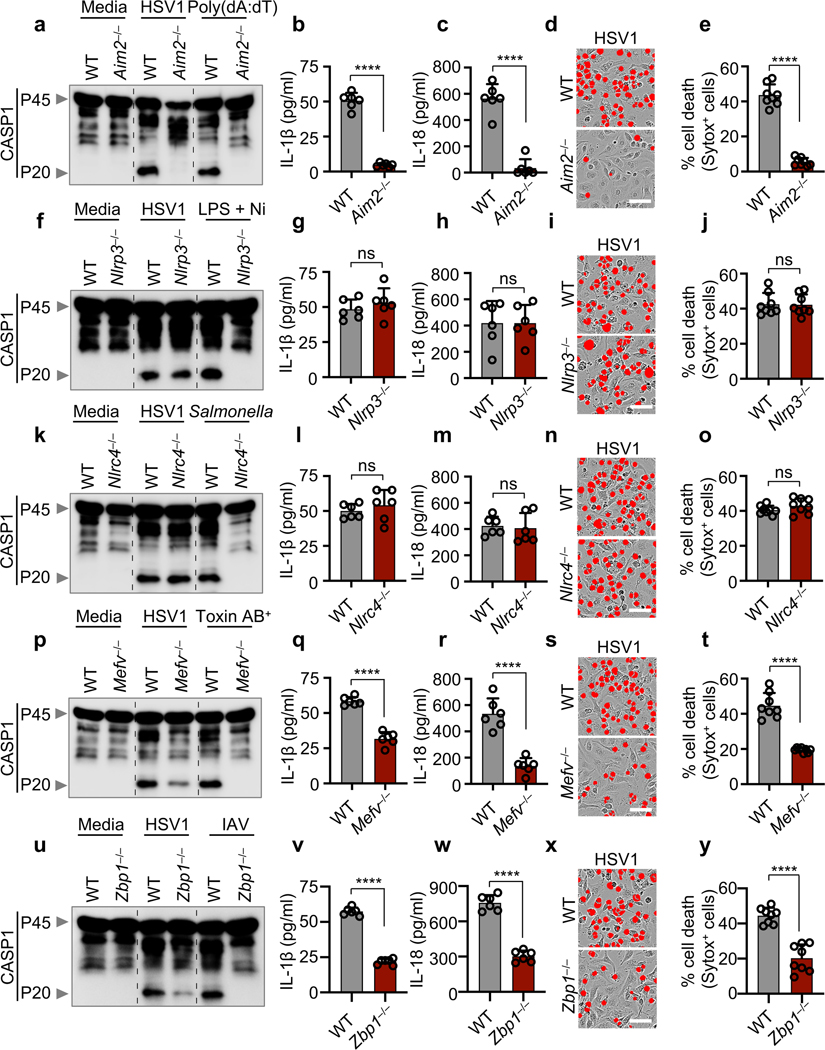
HSV1 induces AIM2-, Pyrin-, ZBP1-mediated caspase-1 activation, cytokine release and cell death. **a,** Immunoblot analysis of pro-caspase-1 (CASP1; P45) and cleaved CASP1 (P20) in HSV1-infected or poly(dA:dT)-transfected wild type (WT) or *Aim2*^*−/−*^ bone marrow-derived macrophages (BMDMs). **b, c,** IL-1β (**b**) and IL-18 (**c**) release following HSV1 infection. **d,** Cell death in BMDMs after HSV1 infection for 16 h. Red indicates dead cells. **e**, Quantification of the cell death in (**d**). **f–j,** Immunoblot analysis of CASP1 (**f**), release of IL-1β (**g**) and IL-18 (**h**), cell death images at 16 h post-infection (**i**), and cell death quantification (**j**) from WT or *Nlrp3*^*−/−*^ BMDMs after HSV1 infection or lipopolysaccharide plus nigericin (LPS + Ni) treatment. **k–o,** Immunoblot analysis of CASP1 (**k**), release of IL-1β (**l**) and IL-18 (**m**), cell death images at 16 h post-infection (**n**), and cell death quantification (**o**) from WT or *Nlrc4*^*−/−*^ BMDMs after HSV1 or *Salmonella* Typhimurium infection. **p–t,** Immunoblot analysis of CASP1 (**p**), release of IL-1β (**q**) and IL-18 (**r**), cell death images at 16 h post-infection (**s**), and cell death quantification (**t**) from WT or *Mefv*^*−/−*^ BMDMs after HSV1 infection or *C. difficile* Toxin AB^+^ supernatant treatment. **u–y,** Immunoblot analysis of CASP1 (**u**), release of IL-1β (**v**) and IL-18 (**w**), cell death images at 16 h post-infection (**x**), and cell death quantification (**y**) from WT or *Zbp1*^*−/−*^ BMDMs after HSV1 or IAV infection. **a,f,k,p,u,** Data are representative of at least three independent experiments. **b,c,g,h,I,m,q,r,v,w,** Data are mean ± s.e.m. ns, not significant; ****P < 0.0001 (two-tailed t-test; *n* = 6 from 3 biologically independent samples). **d,i,n,s,x,** Images are representative of at least three independent experiments. Scale bar, 50 μm. **e,j,o,t,y,** Data are mean ± s.e.m. ns, not significant; ****P < 0.0001 (two-tailed t-test; *n* = 8 from 4 biologically independent samples). Exact *P* values are presented in Supplementary Table 1. For gel source data, see Supplementary Figure 1.

**Fig. 2.**
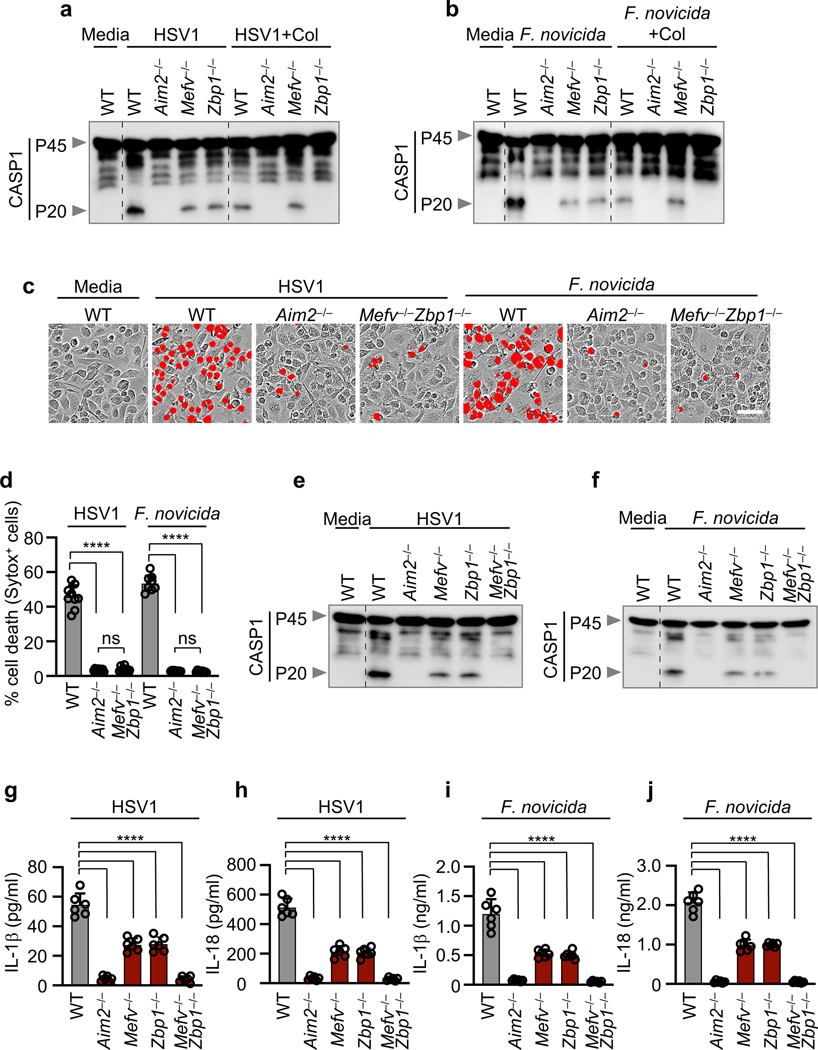
ZBP1 cooperates with Pyrin to drive AIM2-mediated caspase-1 activation, cytokine release and cell death. **a, b,** Immunoblot analysis of caspase-1 (CASP1) in bone marrow-derived macrophages (BMDMs) infected with HSV1 or *F. novicida* with or without colchicine (Col). Data are representative of at least three independent experiments. **c,** Cell death in wild type (WT), *Aim2*^*−/−*^ or *Mefv*^*−/−*^ *Zbp1*^*−/−*^ BMDMs at 16 h post-infection with HSV1 or *F. novicida*. Red indicates dead cells. Data are representative of at least three independent experiments. Scale bar, 50 μm. **d,** Quantification of the cell death from (c). Data are mean ± s.e.m. ns, not significant; ****P < 0.0001 (one-way ANOVA with Dunnett’s multiple comparisons test; *n* = 9 from 3 biologically independent samples). **e, f,** Immunoblot analysis of CASP1 in BMDMs infected with HSV1 (**e**) or *F. novicida* (**f**). Data are representative of at least three independent experiments. **g–j,** Release of IL-1β (**g, i**) or IL-18 (**h, j**) following HSV1 (**g, h**) or *F. novicida* (**i, j**) infection. Data are mean ± s.e.m. ****P < 0.0001 (one-way ANOVA with Dunnett’s multiple comparisons test; *n* = 6 from 3 biologically independent samples). Exact *P* values are presented in Supplementary Table 1. For gel source data, see Supplementary Figure 1.

**Fig. 3.**
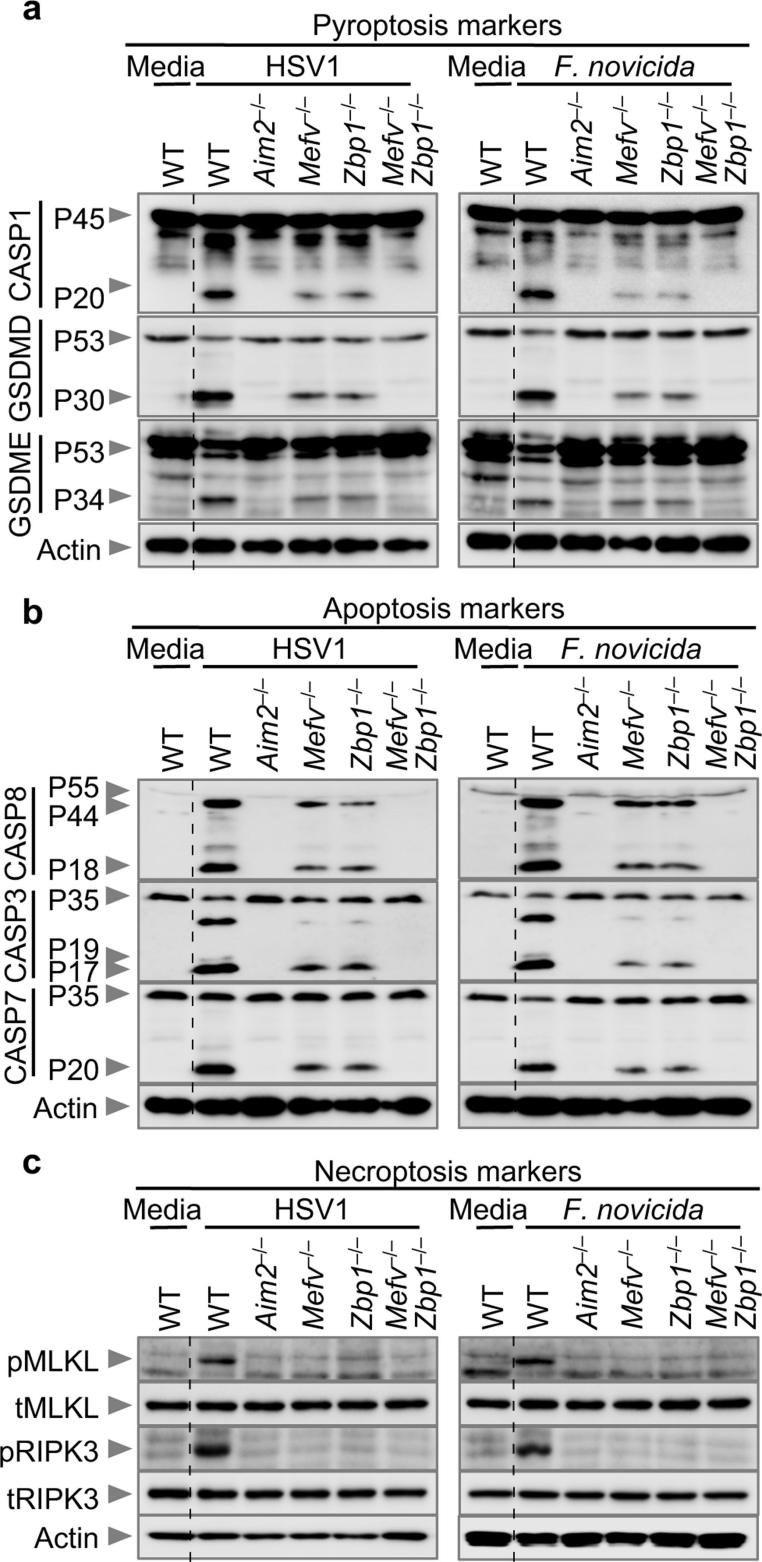
AIM2, Pyrin and ZBP1 promote inflammatory cell death in response to HSV1 and *F. novicida* infections. **a–c,** Immunoblot analysis of (**a**) pro- (P45) and activated (P20) caspase-1 (CASP1), pro- (P53) and activated (P30) gasdermin D (GSDMD), pro- (P53) and activated (P34) gasdermin E (GSDME); (**b**) pro- (P55) and cleaved (P18) caspase-8 (CASP8), pro- (P35) and cleaved (P17/P19) caspase-3 (CASP3), pro- (P35) and cleaved (P20) caspase-7 (CASP7); (**c**) phosphorylated MLKL (pMLKL), total MLKL (tMLKL), phosphorylated RIPK3 (pRIPK3) and total RIPK3 (tRIPK3) in wild type (WT), *Aim2*^*−/−*^*, Mefv*^*−/−*^*, Zbp1*^*−/−*^ or *Mefv*^*−/−*^*Zbp1*^*−/−*^ bone marrow-derived macrophages (BMDMs) after HSV1 or *F. novicida* infection. Data are representative of at least three independent experiments. For gel source data, see Supplementary Figure 1.

**Fig. 4.**
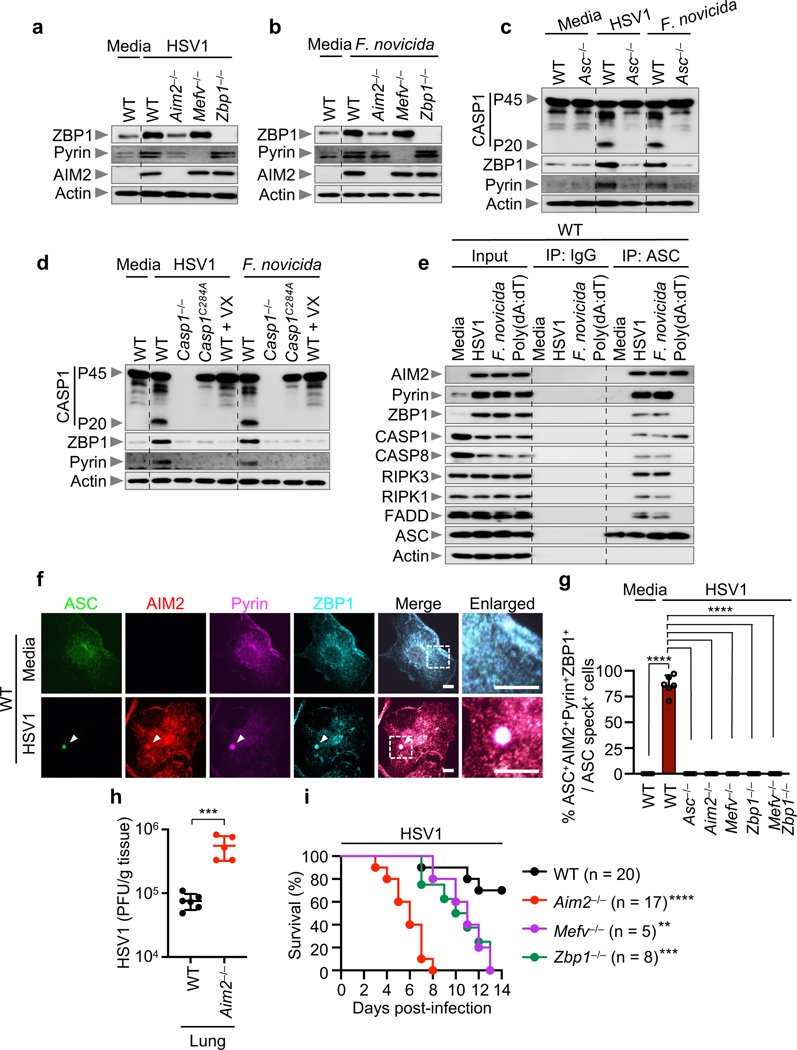
AIM2-mediated signaling acts as an upstream regulator of Pyrin and ZBP1, which are required to form the AIM2 PANoptosome. **a, b,** Immunoblot analysis of ZBP1, Pyrin and AIM2 in wild type (WT), *Aim2*^*−/−*^*, Mefv*^*−/−*^ and *Zbp1*^*−/−*^ bone marrow-derived macrophages (BMDMs) infected with HSV1 (**a**) or *F. novicida* (**b**). **c,** Immunoblot analysis of caspase-1 (CASP1) activation and Pyrin and ZBP1 expression in WT or *Asc*^*−/−*^ BMDMs after HSV1 or *F. novicida* infection. **d,** Immunoblot analysis of CASP1 activation and Pyrin and ZBP1 expression in WT, *Casp1*^*−/−*^ and *Casp1*^*C284A/C284A*^ (*Casp1*^*C284A*^) BMDMs and WT BMDMs treated with CASP1 inhibitor VX-765 (20 μM; VX) after HSV1 or *F. novicida* infection. **e,** Immunoprecipitation (IP) in WT BMDMs with IgG control antibodies or anti-ASC antibodies after HSV1 or *F. novicida* infection or poly(dA:dT) transfection. Data are representative of at least three independent experiments (**a**–**e**). **f,** Immunofluorescence images of WT BMDMs at 12 h after HSV1 infection. Scale bars, 5 μm. Arrowheads indicate the ASC speck. Images are representative of three independent experiments. **g,** Quantification of the percentage of cells with ASC^+^AIM2^+^Pyrin^+^ZBP1^+^ specks among the ASC speck^+^ cells. Data are mean ± s.e.m. ****P < 0.0001 (one-way ANOVA with Dunnett’s multiple comparisons test; *n* = 6 from 3 biologically independent samples). **h,** Pulmonary viral titer at day 3 after infection with HSV1 (WT, *n* = 6*; Aim2*^*−/−*^, *n* = 5). Each symbol represents one mouse. Data are pooled from two independent experiments. Data are mean ± s.e.m. ***P < 0.001 (two-tailed t-test). **i,** Survival of WT, *Aim2*^*−/−*^, *Mefv*^*−/−*^ and *Zbp1*^*−/−*^ mice infected intranasally with 5 × 10^7^ PFU HSV1. Survival data are pooled from three independent experiments. *P* values for survival of each genotype vs WT are shown in the key. **P < 0.01; ***P < 0.001; ****P < 0.0001 (log-rank (Mantel-Cox) test). Exact *P* values are presented in Supplementary Table 1. For gel source data, see Supplementary Figure 1.
