# Supplementary material for: AIM2 forms a complex with Pyrin and ZBP1 to drive PANoptosis and host defense
Source: Nature. Author manuscript; Available in PMC 2022 Sep 1. (PMC8603942; doi:10.1038/s41586-021-03875-8)
Supplement: Supplementary Figure 1 [file NIHMS1739724-supplement-Supplementary_Figure_1.pdf]

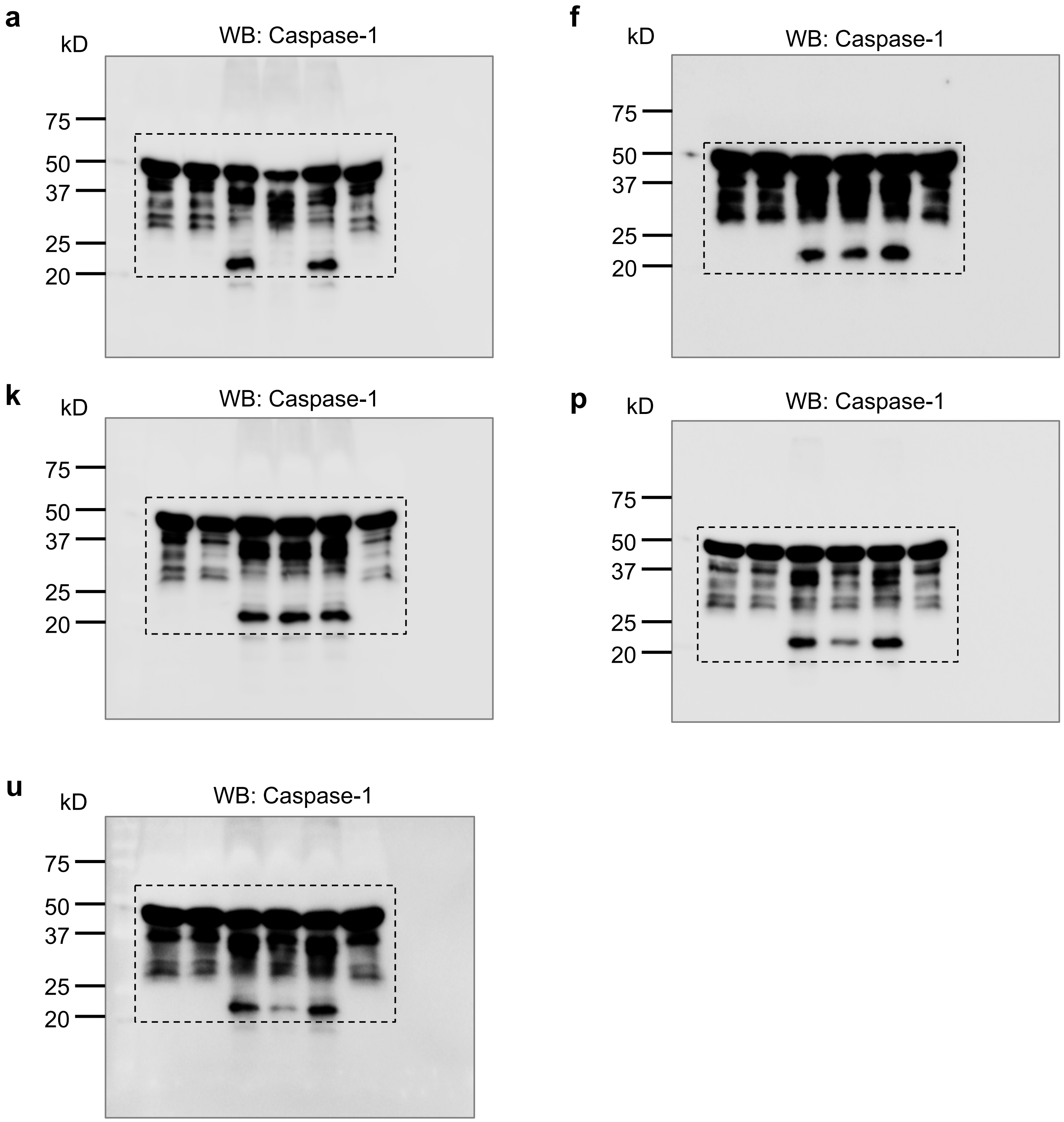

Figure 1

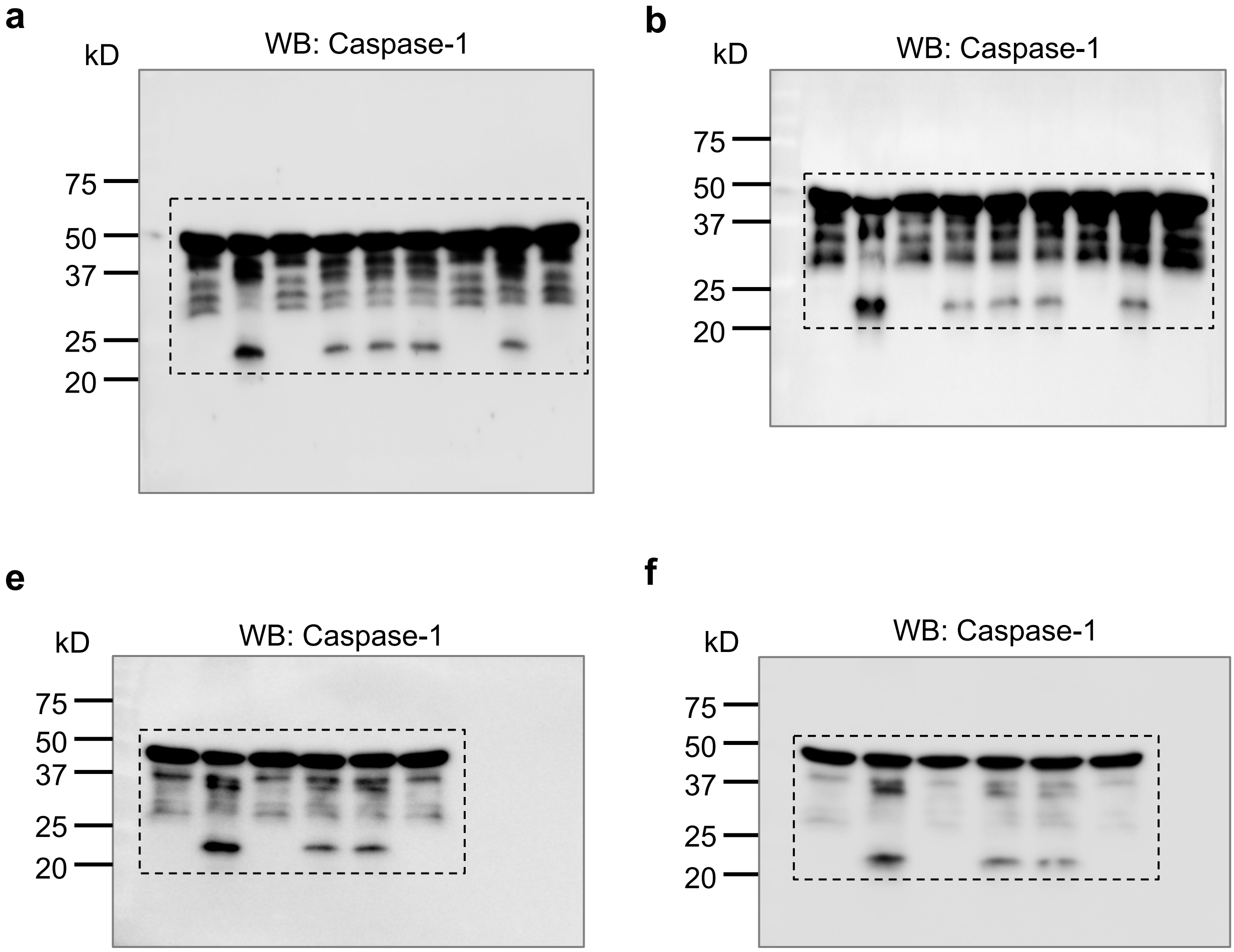
a
WB: Caspase-1
kD
75
50
37
25
20
b
WB: Caspase-1
kD
75
50
37
25
20
e
WB: Caspase-1
kD
75
50
37
25
20
f
WB: Caspase-1
kD
75
50
37
25
20

Figure 2

a

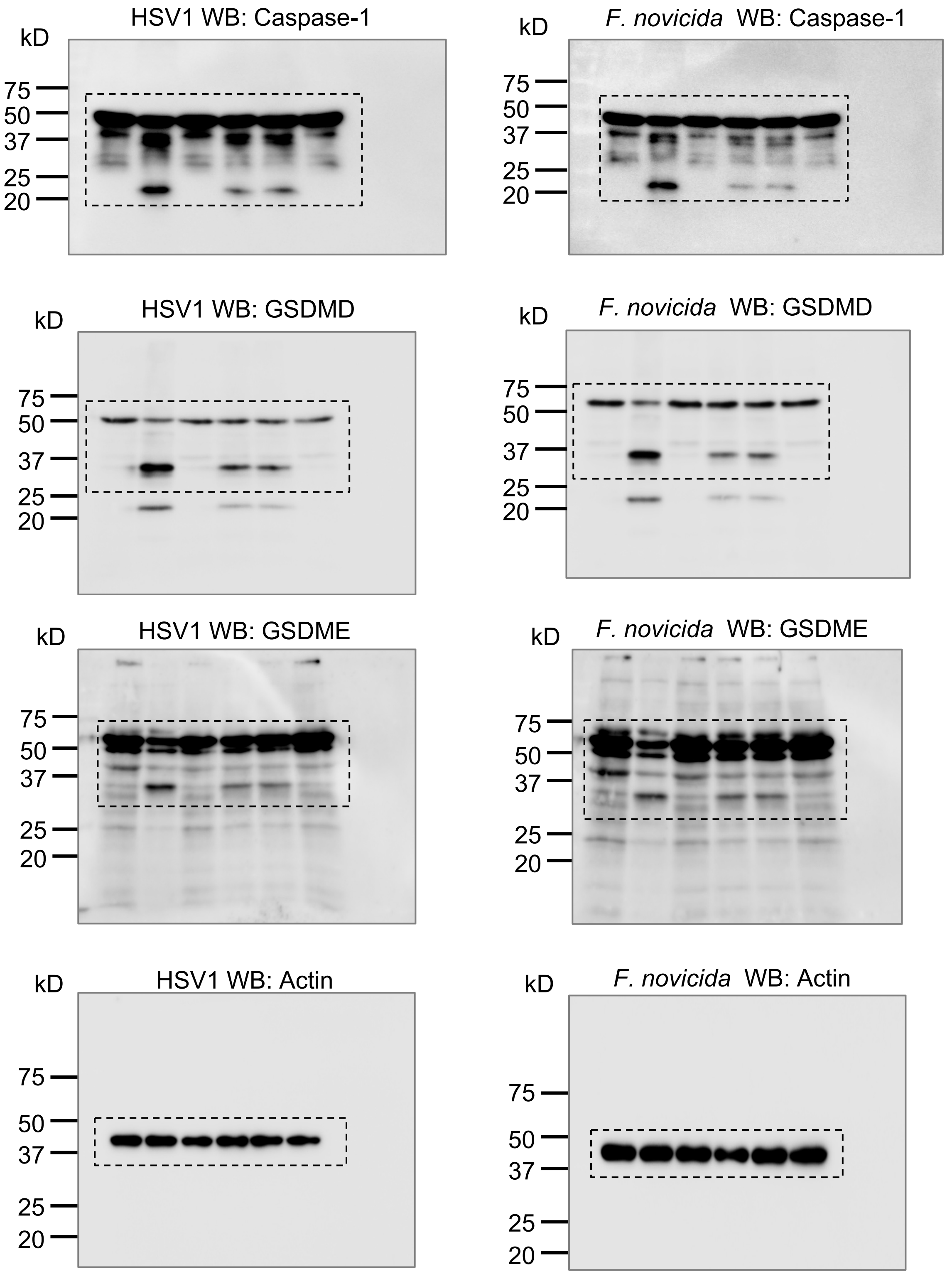
HSV1 WB: Caspase-1
kD
75
50
37
25
20
F. novicida WB: Caspase-1
kD
75
50
37
25
20
HSV1 WB: GSDMD
kD
75
50
37
25
20
F. novicida WB: GSDMD
kD
75
50
37
25
20
HSV1 WB: GSDME
kD
75
50
37
25
20
F. novicida WB: GSDME
kD
75
50
37
25
20
HSV1 WB: Actin
kD
75
50
37
25
20
F. novicida WB: Actin
kD
75
50
37
25
20

Figure 3

b

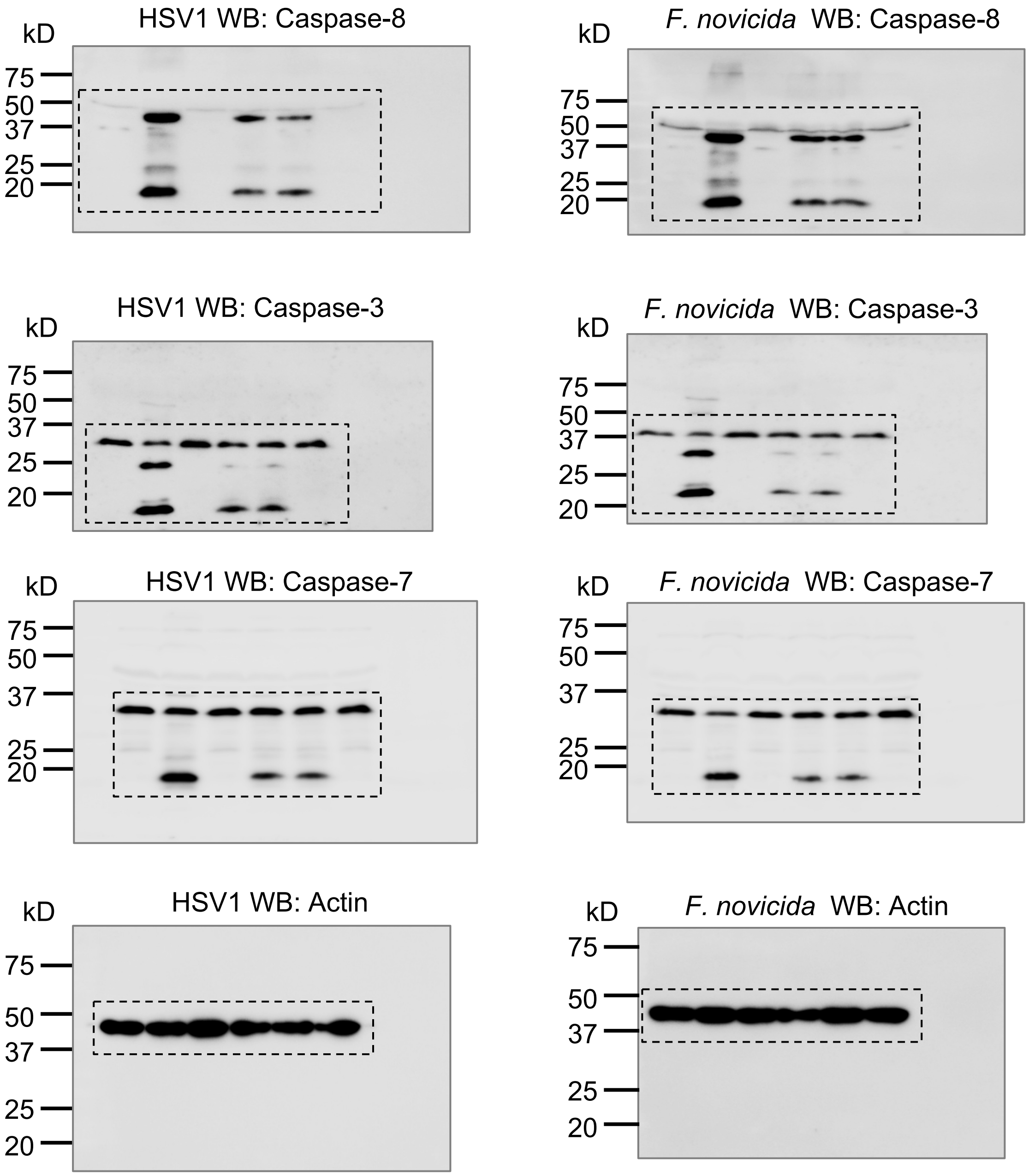
HSV1 WB: Caspase-8
kD
75
50
37
25
20
F. novicida WB: Caspase-8
kD
75
50
37
25
20
HSV1 WB: Caspase-3
kD
75
50
37
25
20
F. novicida WB: Caspase-3
kD
75
50
37
25
20
HSV1 WB: Caspase-7
kD
75
50
37
25
20
F. novicida WB: Caspase-7
kD
75
50
37
25
20
HSV1 WB: Actin
kD
75
50
37
25
20
F. novicida WB: Actin
kD
75
50
37
25
20

Figure 3

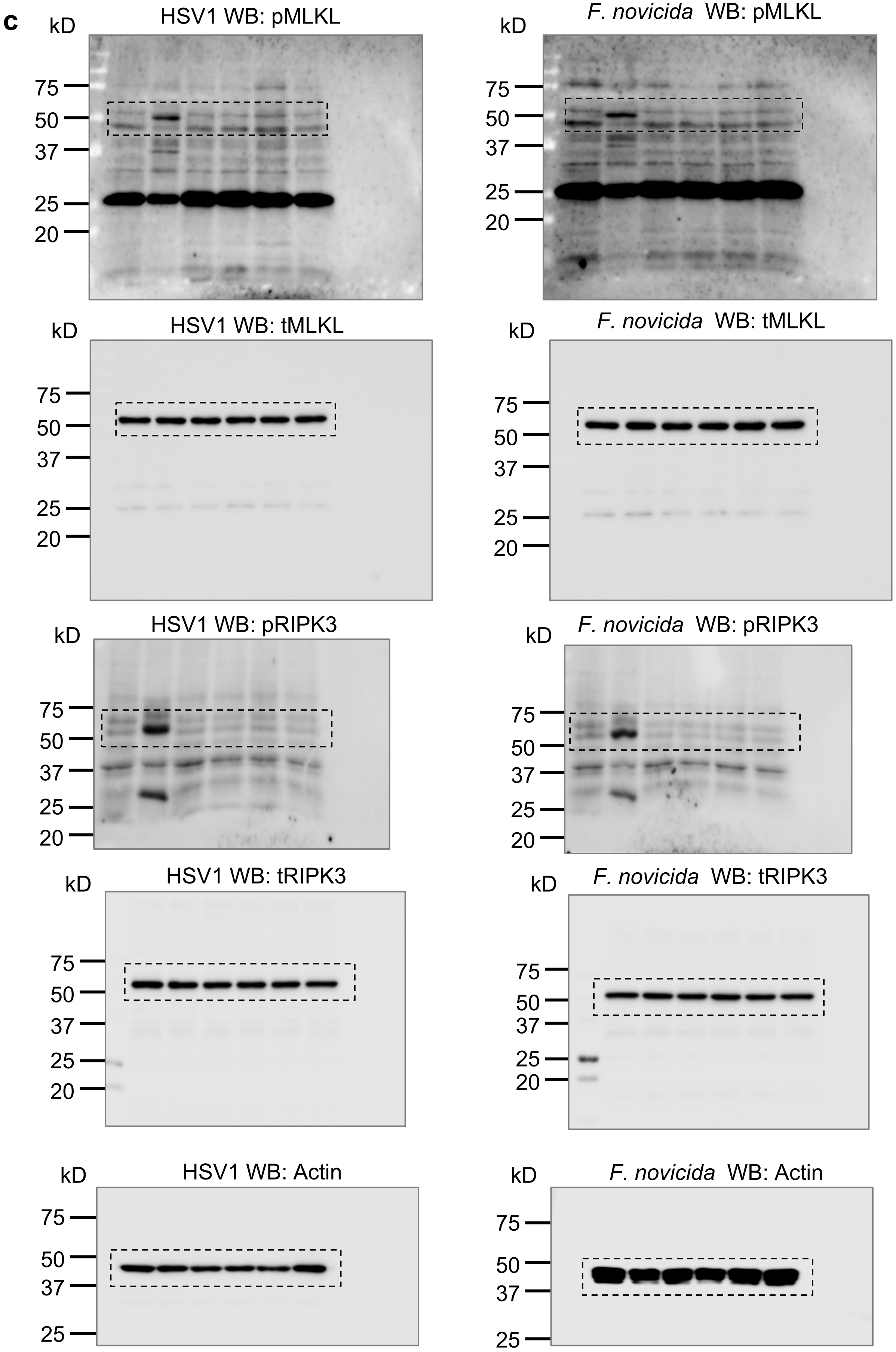
c
HSV1 WB: pMLKL
F. novicida WB: pMLKL
HSV1 WB: tMLKL
F. novicida WB: tMLKL
HSV1 WB: pRIPK3
F. novicida WB: pRIPK3
HSV1 WB: tRIPK3
F. novicida WB: tRIPK3
HSV1 WB: Actin
F. novicida WB: Actin
kD
75
50
37
25
20

Figure 3

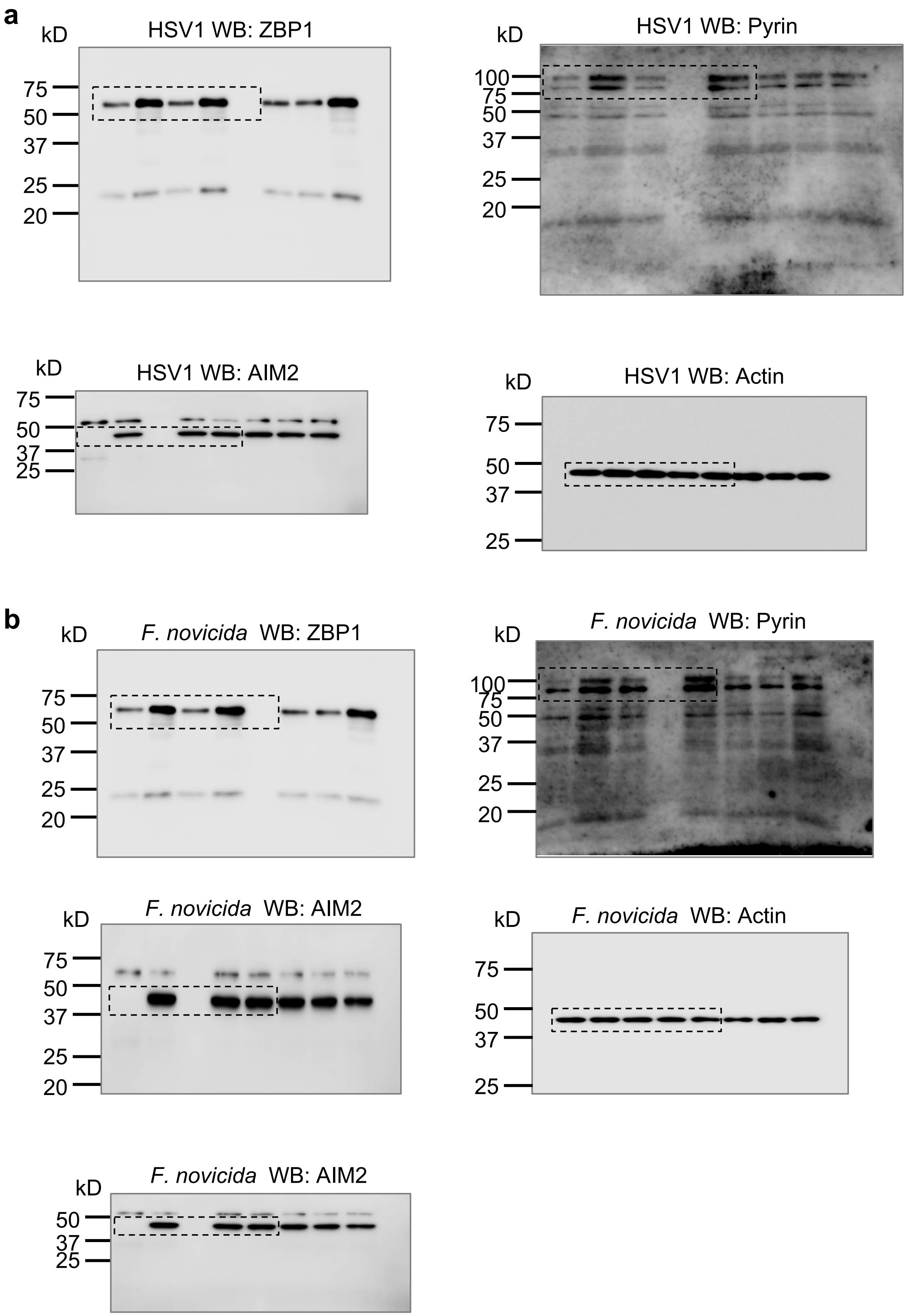
a
kD
HSV1 WB: ZBP1
75
50
37
25
20
HSV1 WB: Pyrin
100
75
50
37
25
20
HSV1 WB: AIM2
75
50
37
25
HSV1 WB: Actin
75
50
37
25
b
F. novicida WB: ZBP1
F. novicida WB: Pyrin
F. novicida WB: AIM2
F. novicida WB: Actin
F. novicida WB: AIM2

Figure 4

c

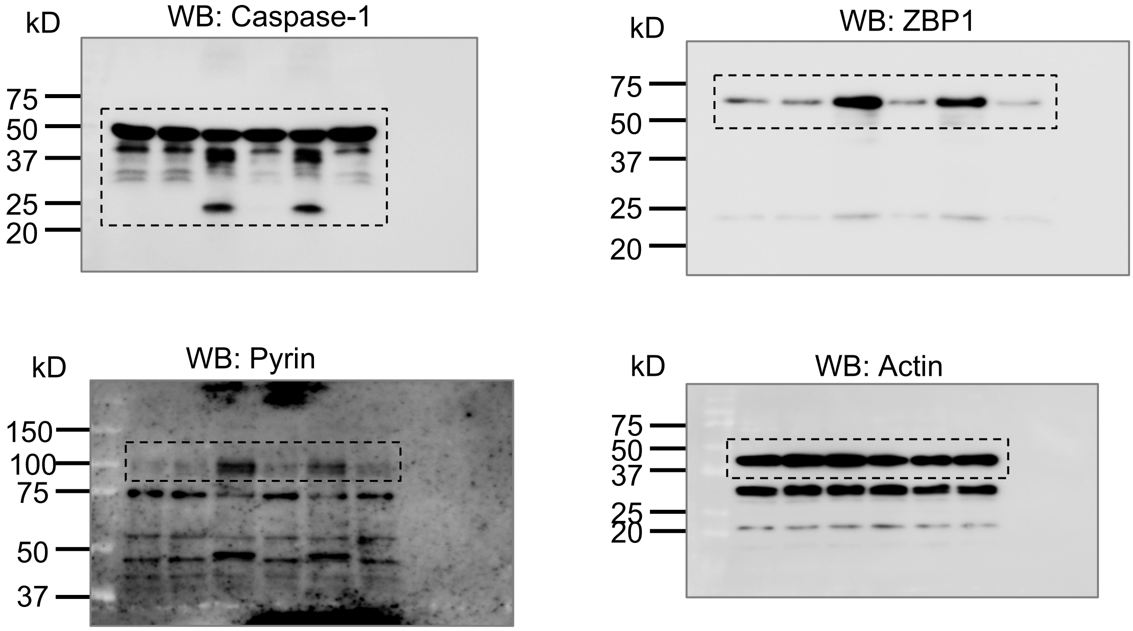

WB: Caspase-1
kD
75
50
37
25
20
WB: ZBP1
kD
75
50
37
25
20
WB: Pyrin
kD
150
100
75
50
37
WB: Actin
kD
75
50
37
25
20

d

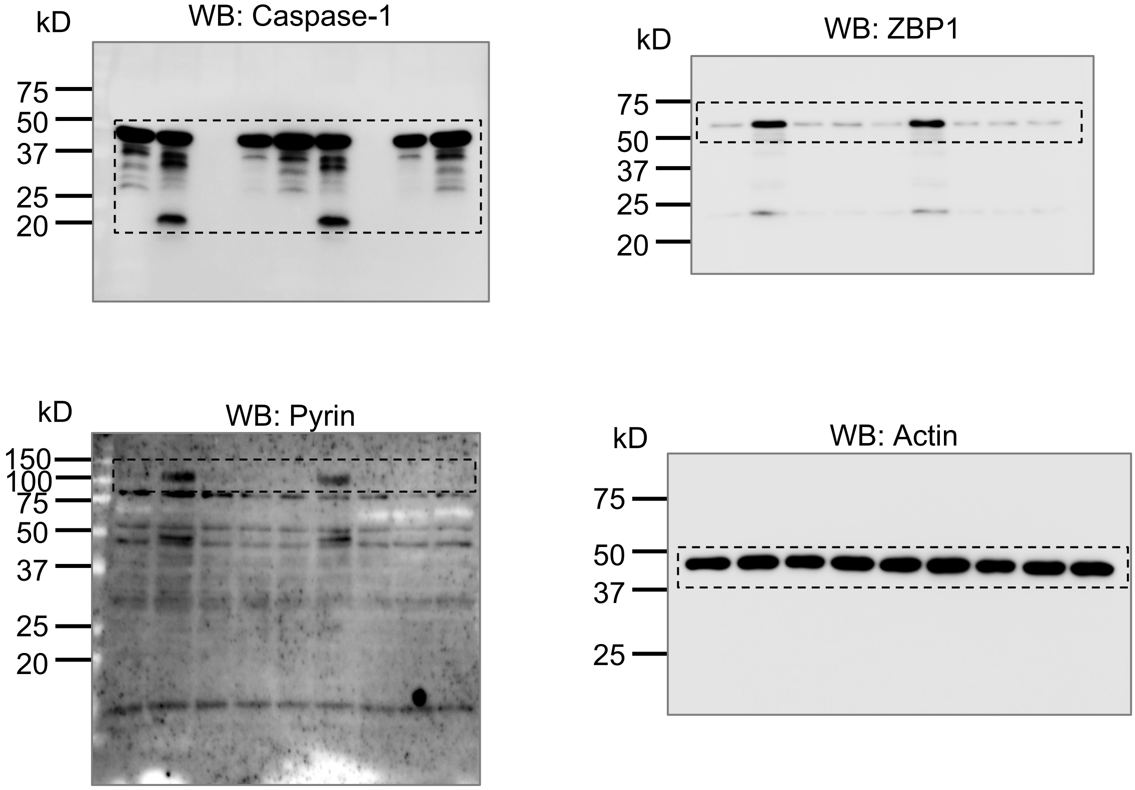

WB: Caspase-1
kD
75
50
37
25
20
WB: ZBP1
kD
75
50
37
25
20
WB: Pyrin
kD
150
100
75
50
37
25
20
WB: Actin
kD
75
50
37
25

Figure 4

e

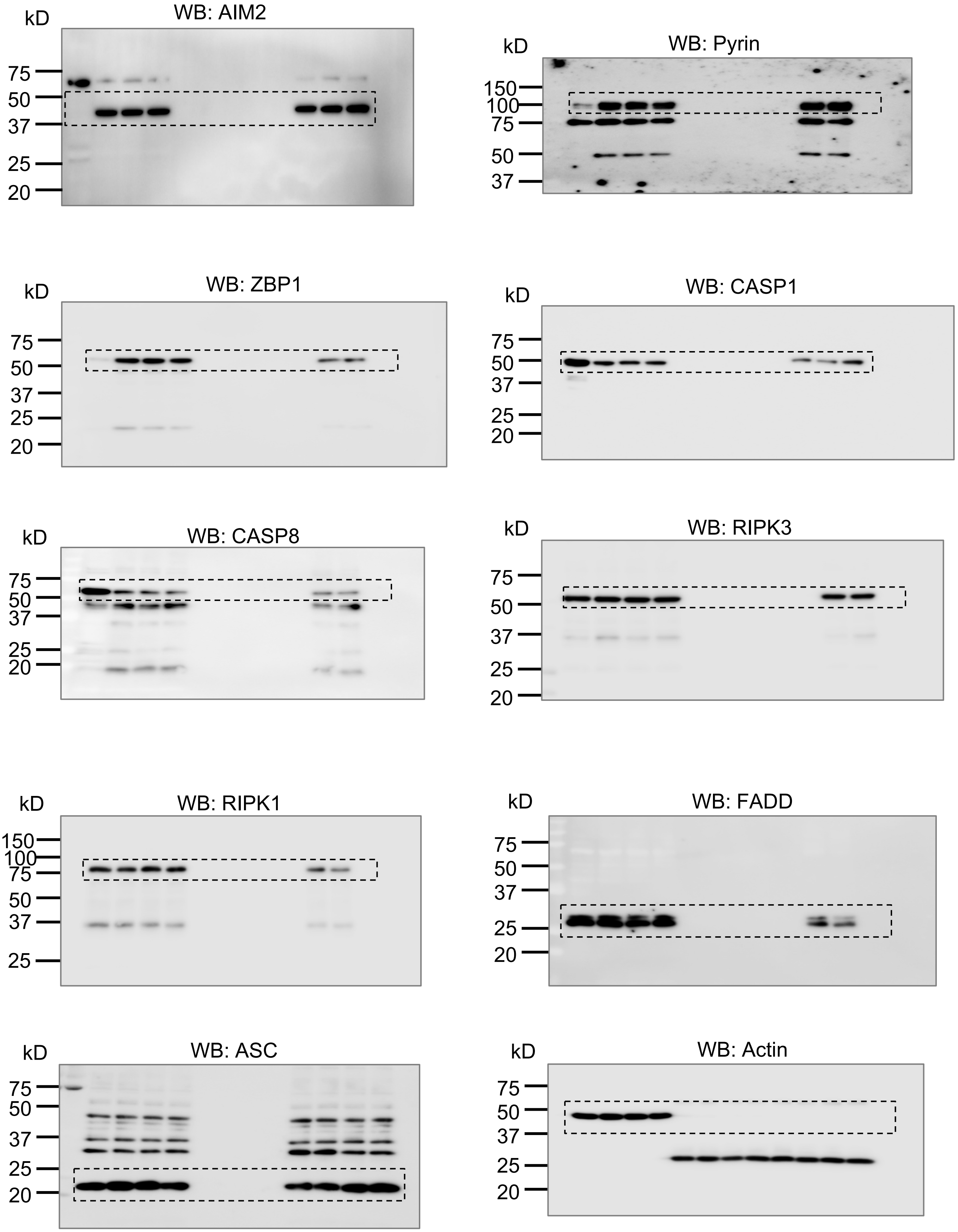

WB: AIM2
kD
75
50
37
25
20
WB: Pyrin
kD
150
100
75
50
37
WB: ZBP1
kD
75
50
37
25
20
WB: CASP1
kD
75
50
37
25
20
WB: CASP8
kD
75
50
37
25
20
WB: RIPK3
kD
75
50
37
25
20
WB: RIPK1
kD
150
100
75
50
37
25
WB: FADD
kD
75
50
37
25
20
WB: ASC
kD
75
50
37
25
20
WB: Actin
kD
75
50
37
25
20

Figure 4

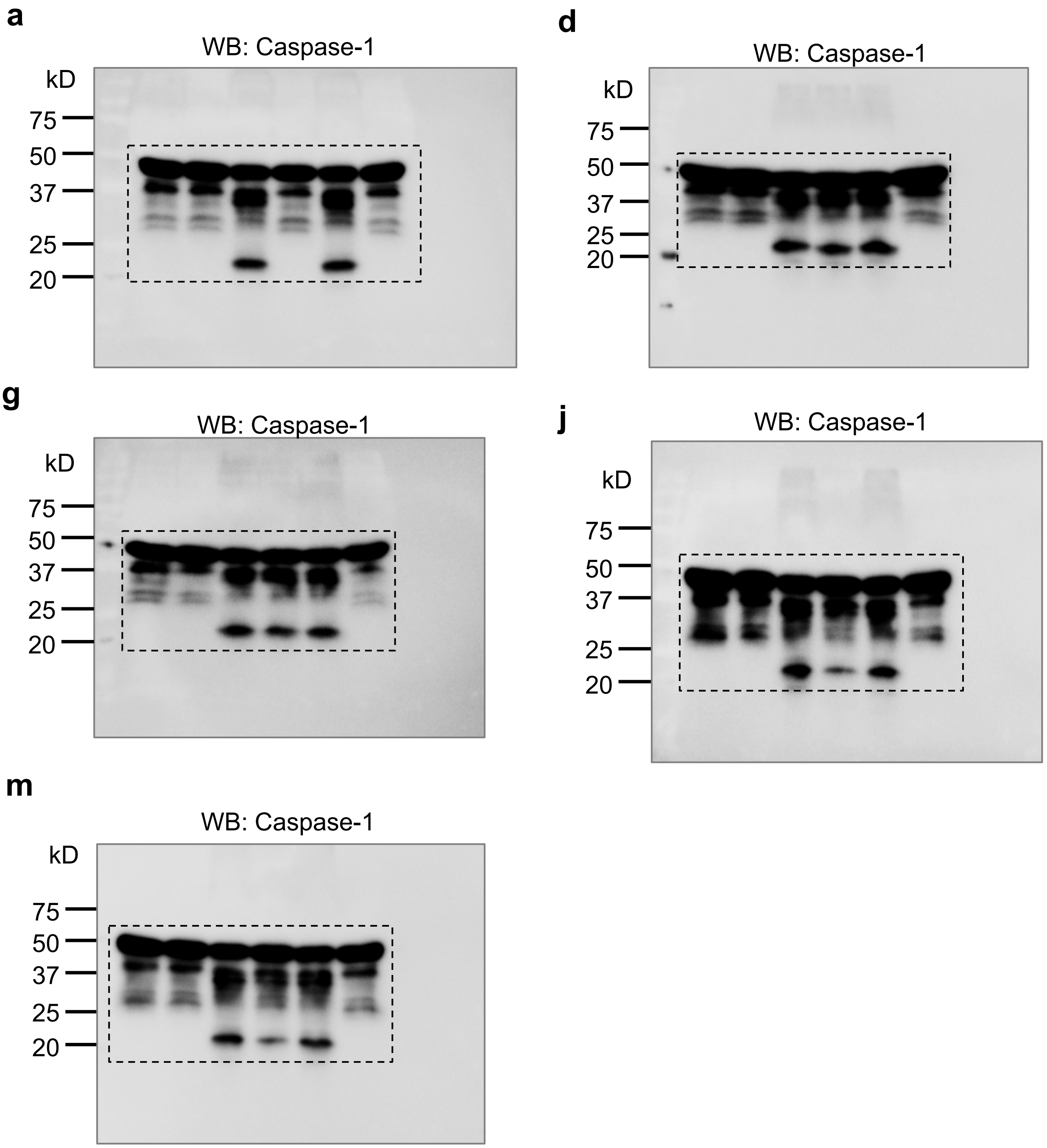

Extended Data Figure 1

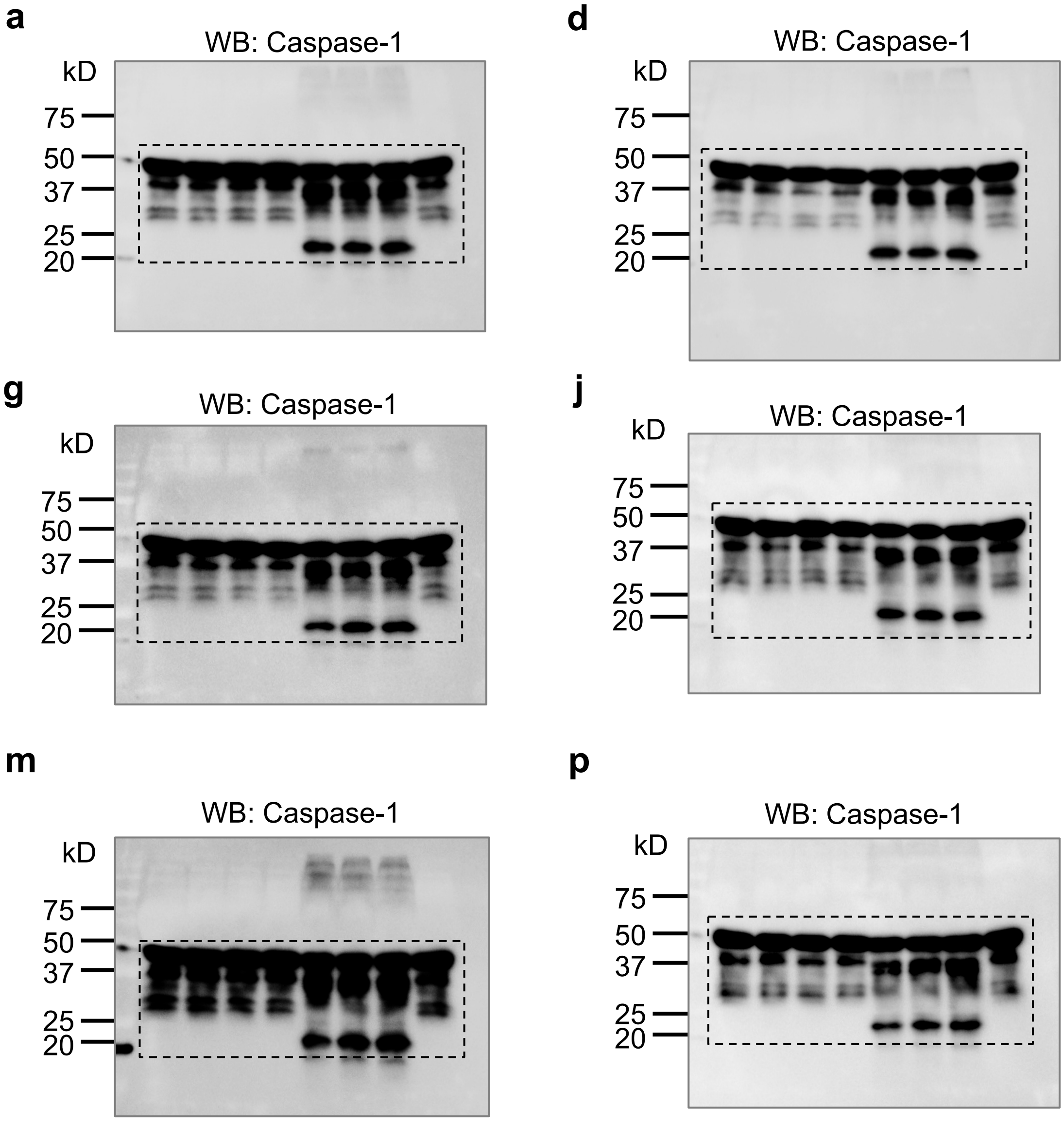

Extended Data Figure 2

e

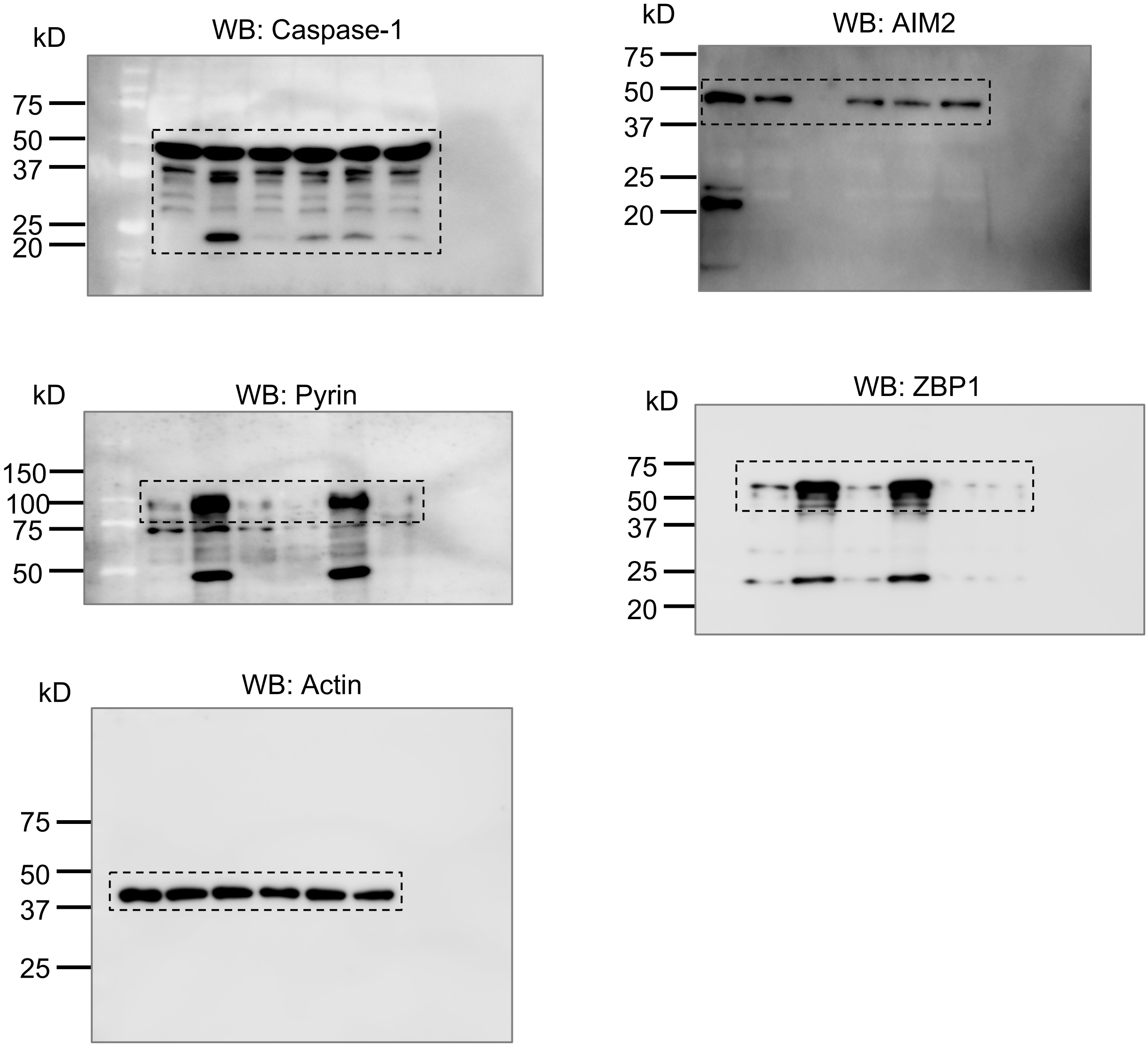

h

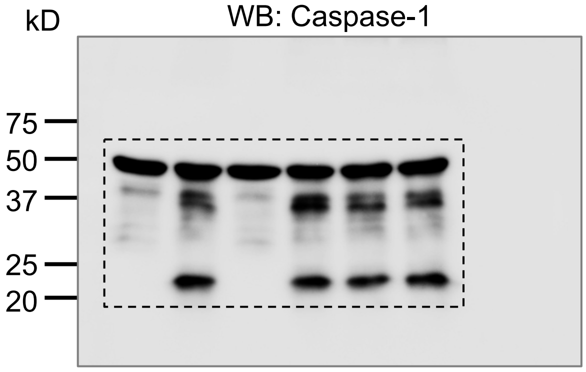

Extended Data Figure 4

b

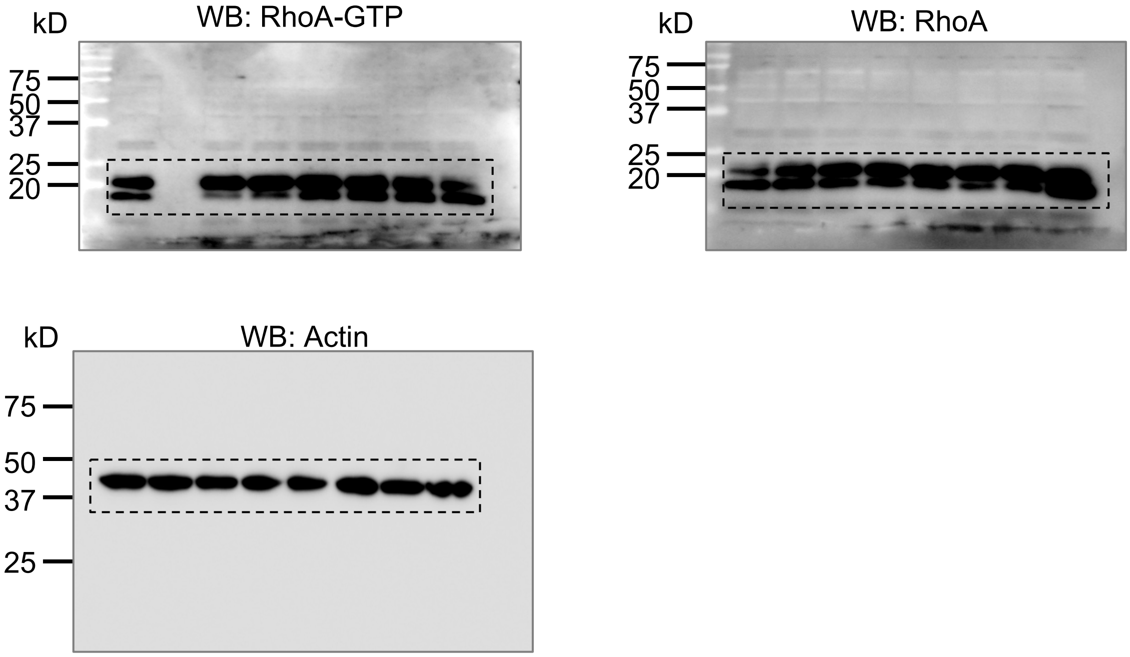

c

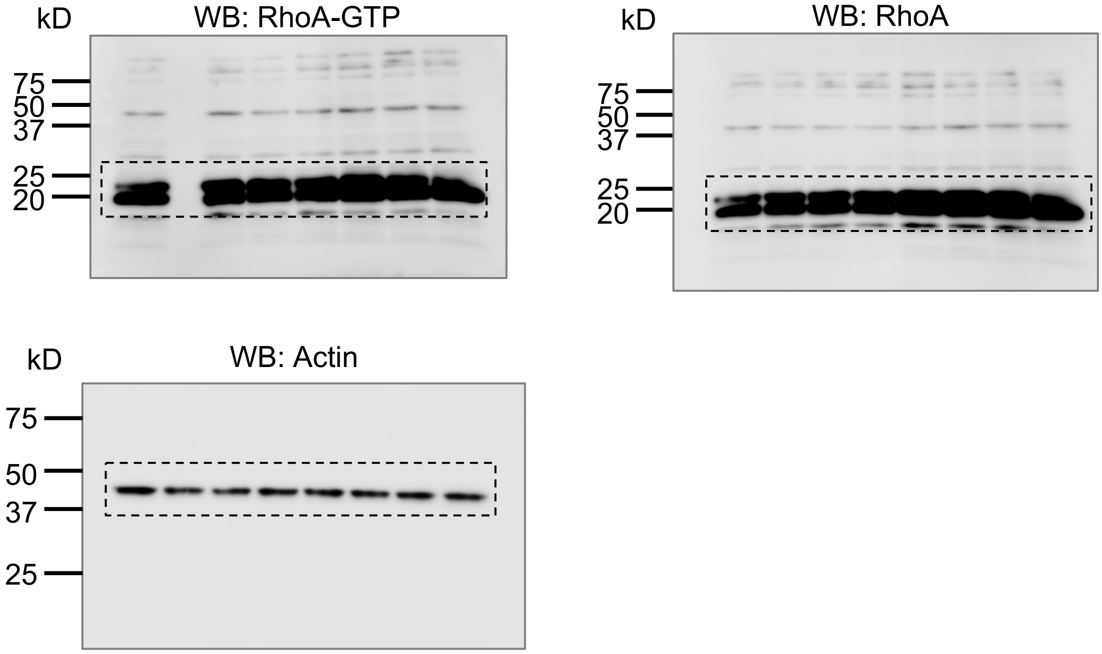

Extended Data Figure 5

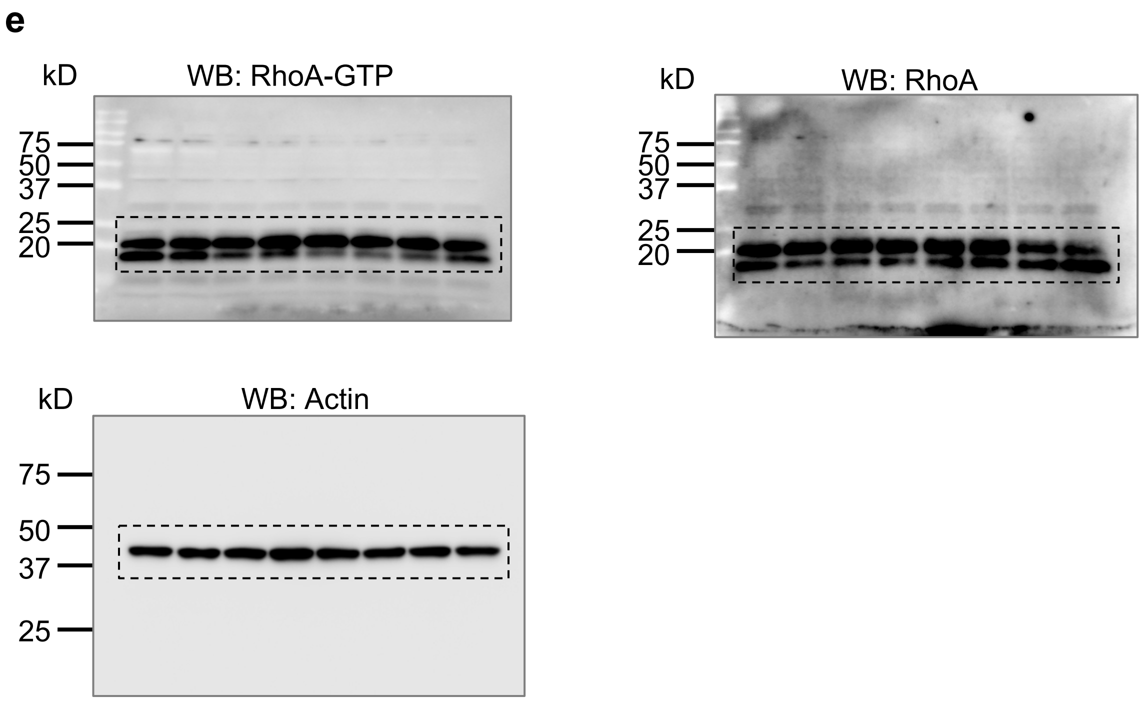
e
kD
WB: RhoA-GTP
75
50
37
25
20
kD
WB: RhoA
75
50
37
25
20
kD
WB: Actin
75
50
37
25

a

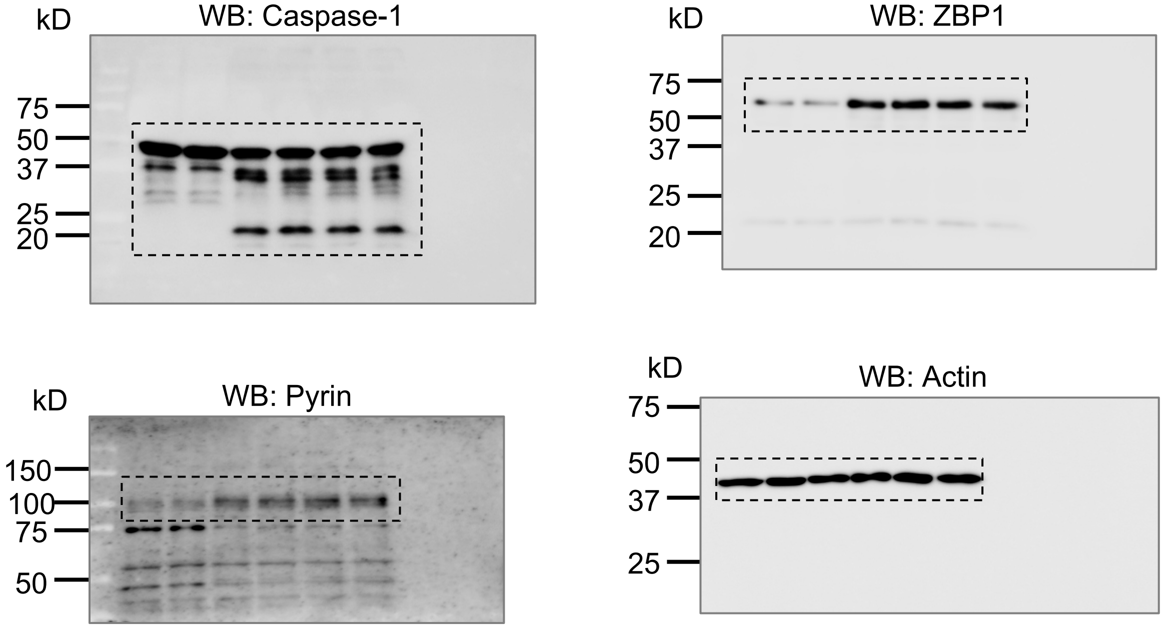

b

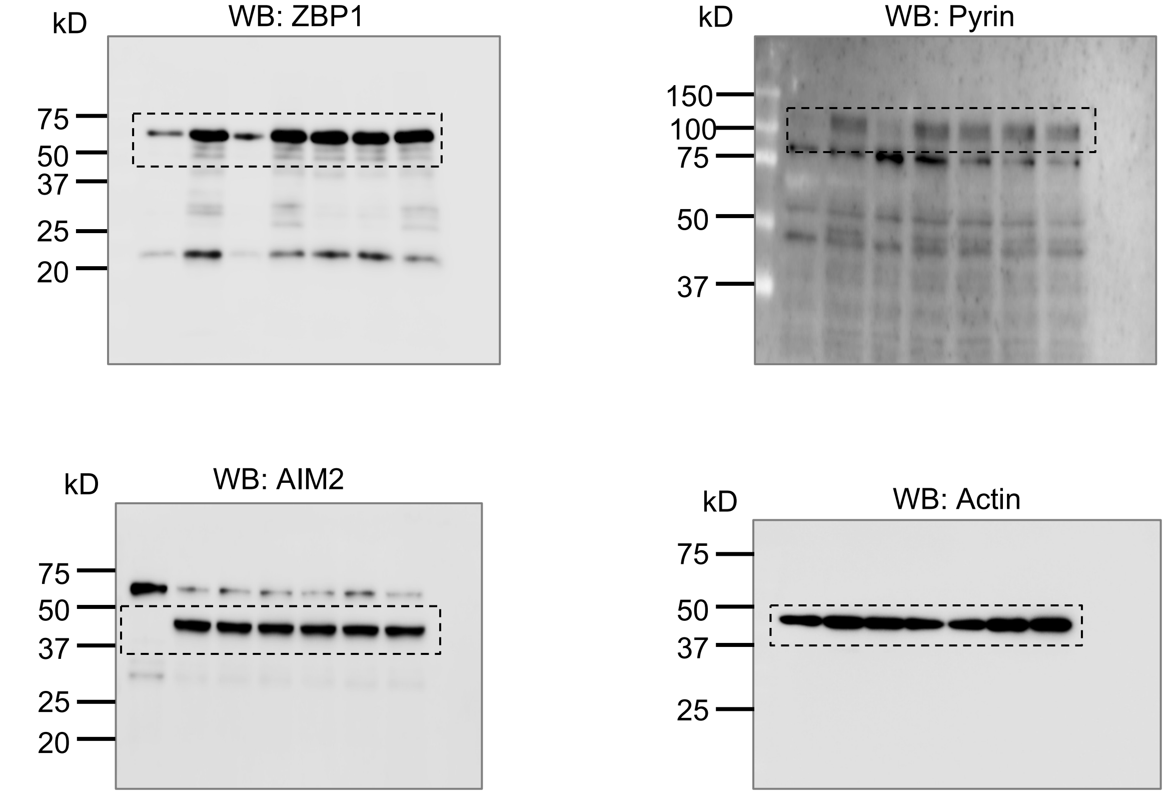

Extended Data Figure 6

c

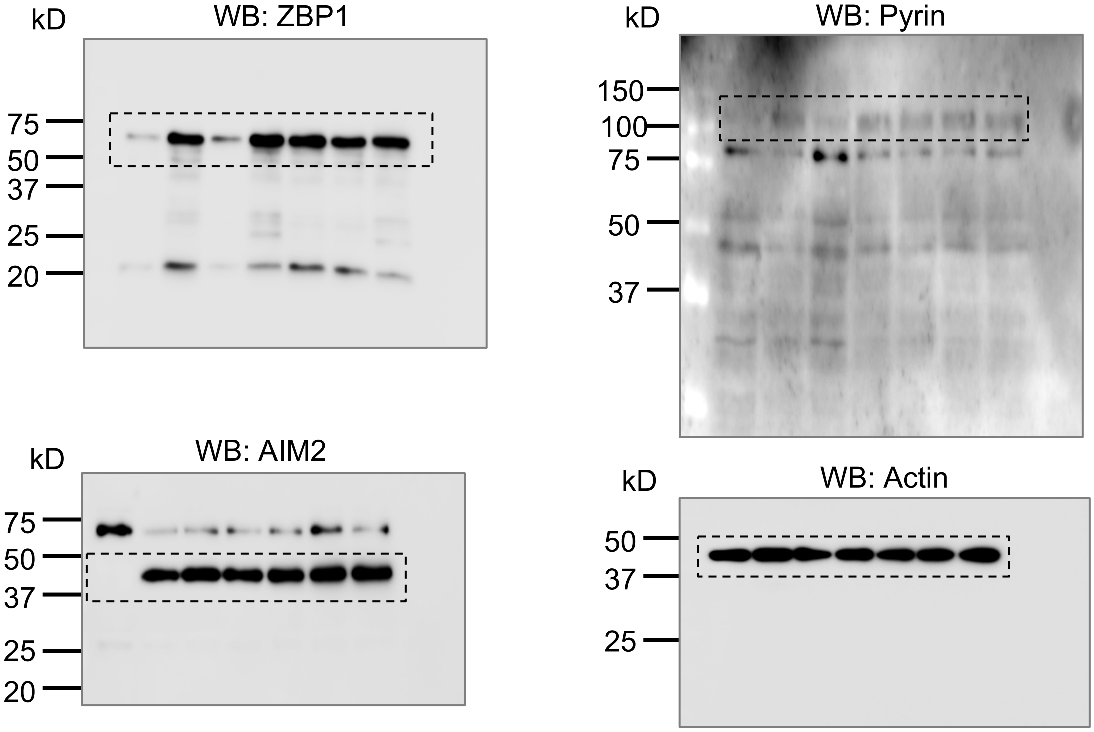

d

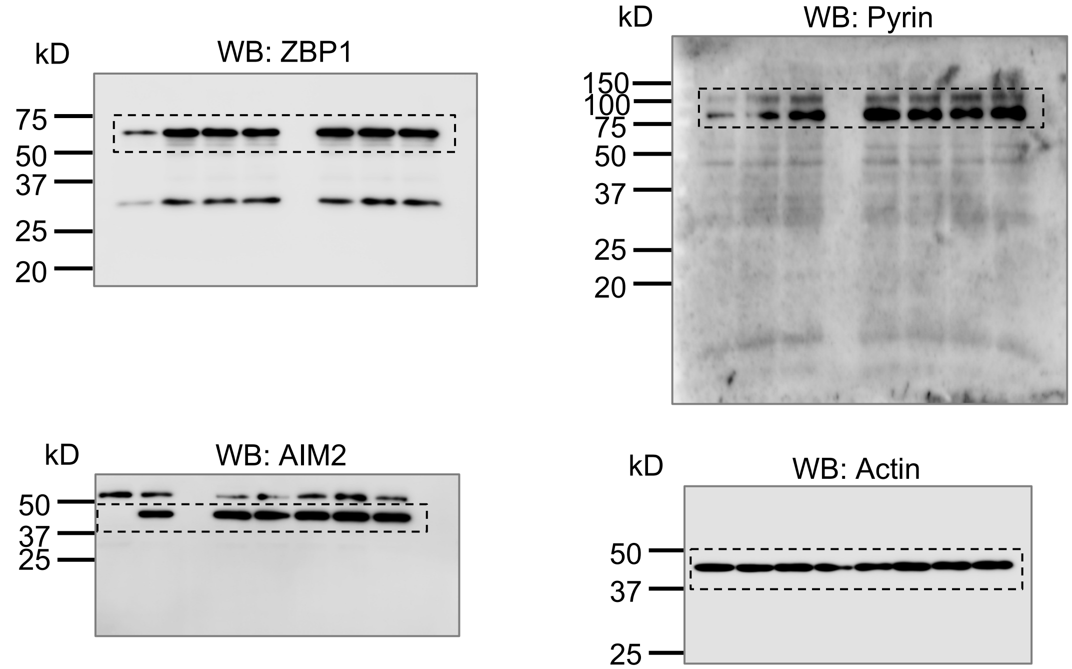

Extended Data Figure 6

g

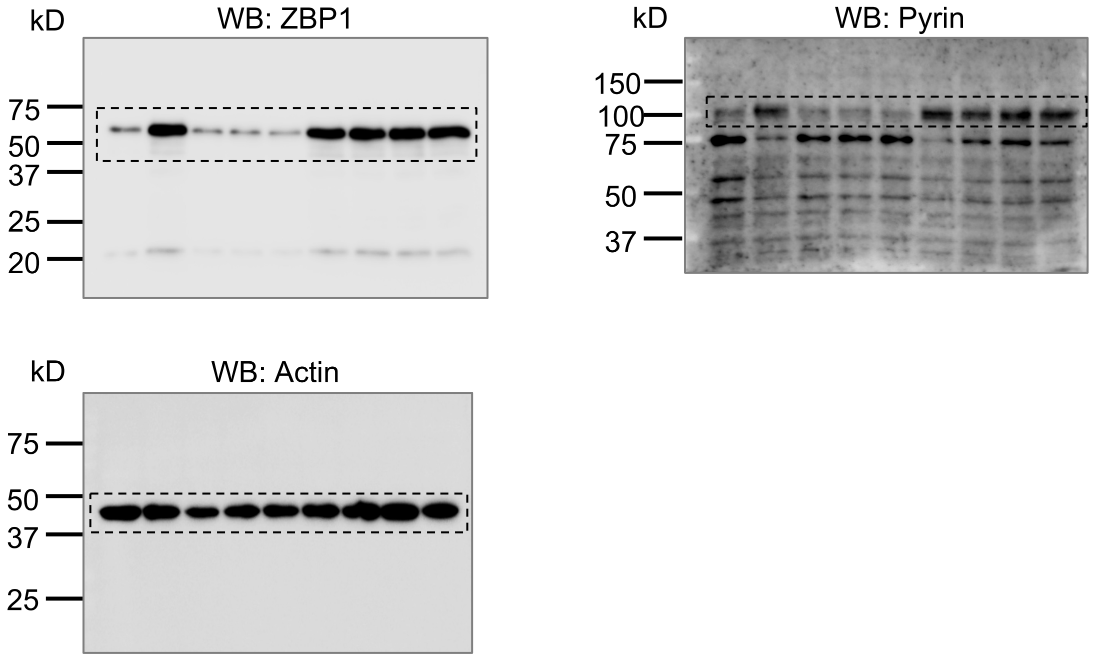

h

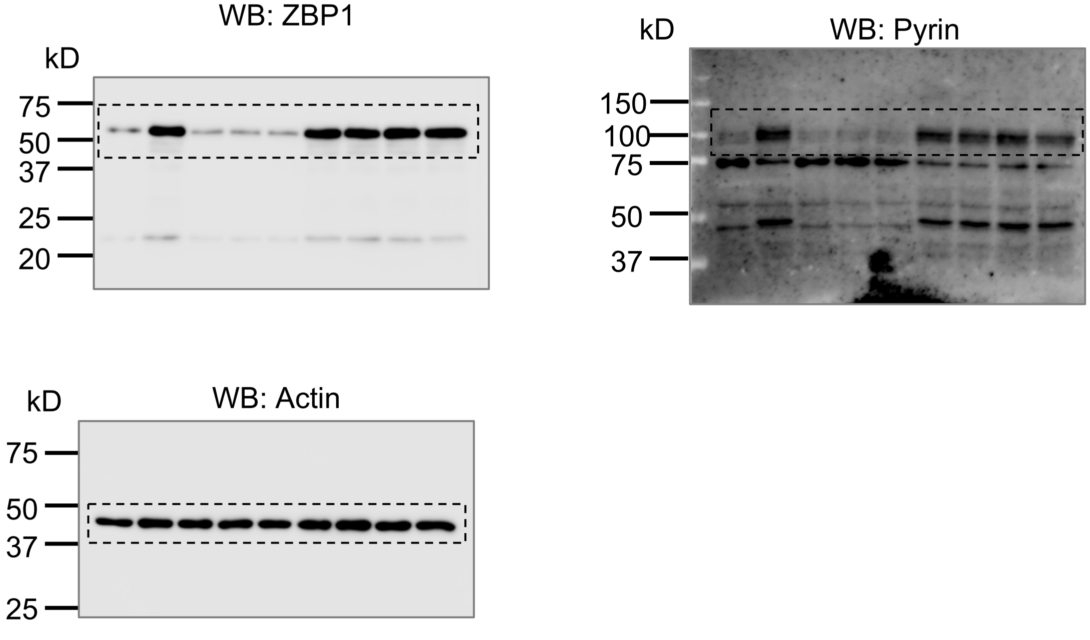

Extended Data Figure 7

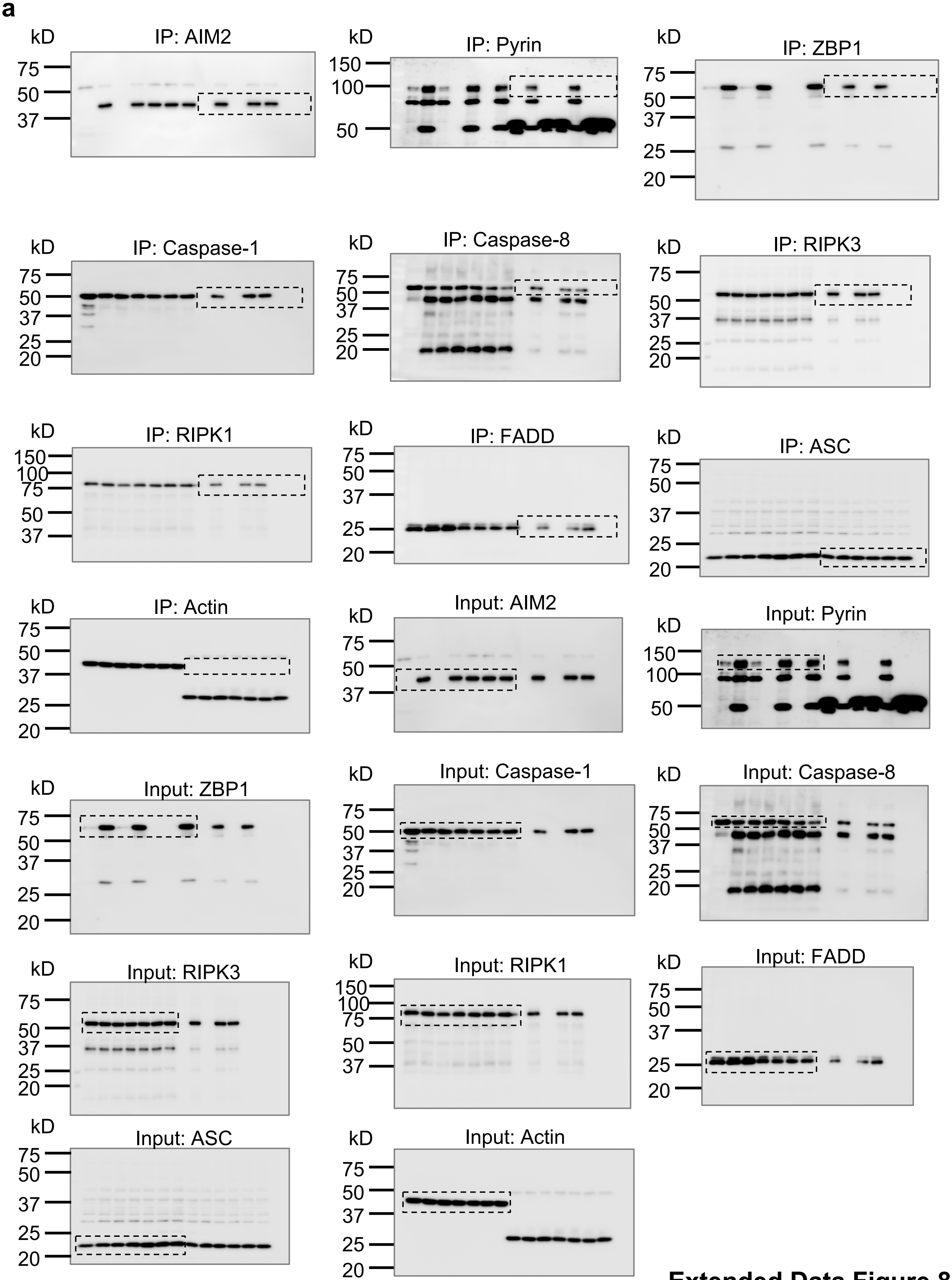

Extended Data Figure 8

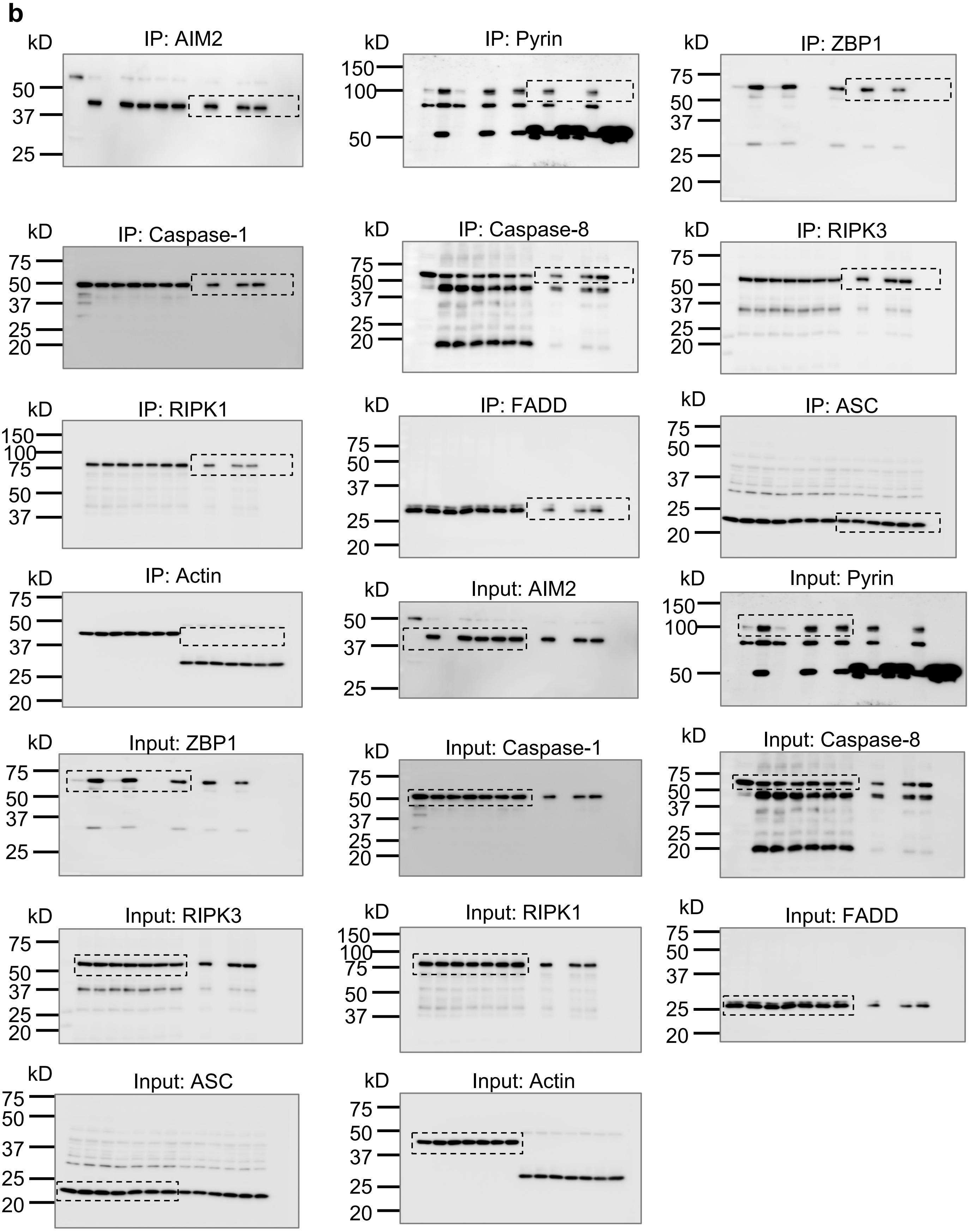

Extended Data Figure 8

c

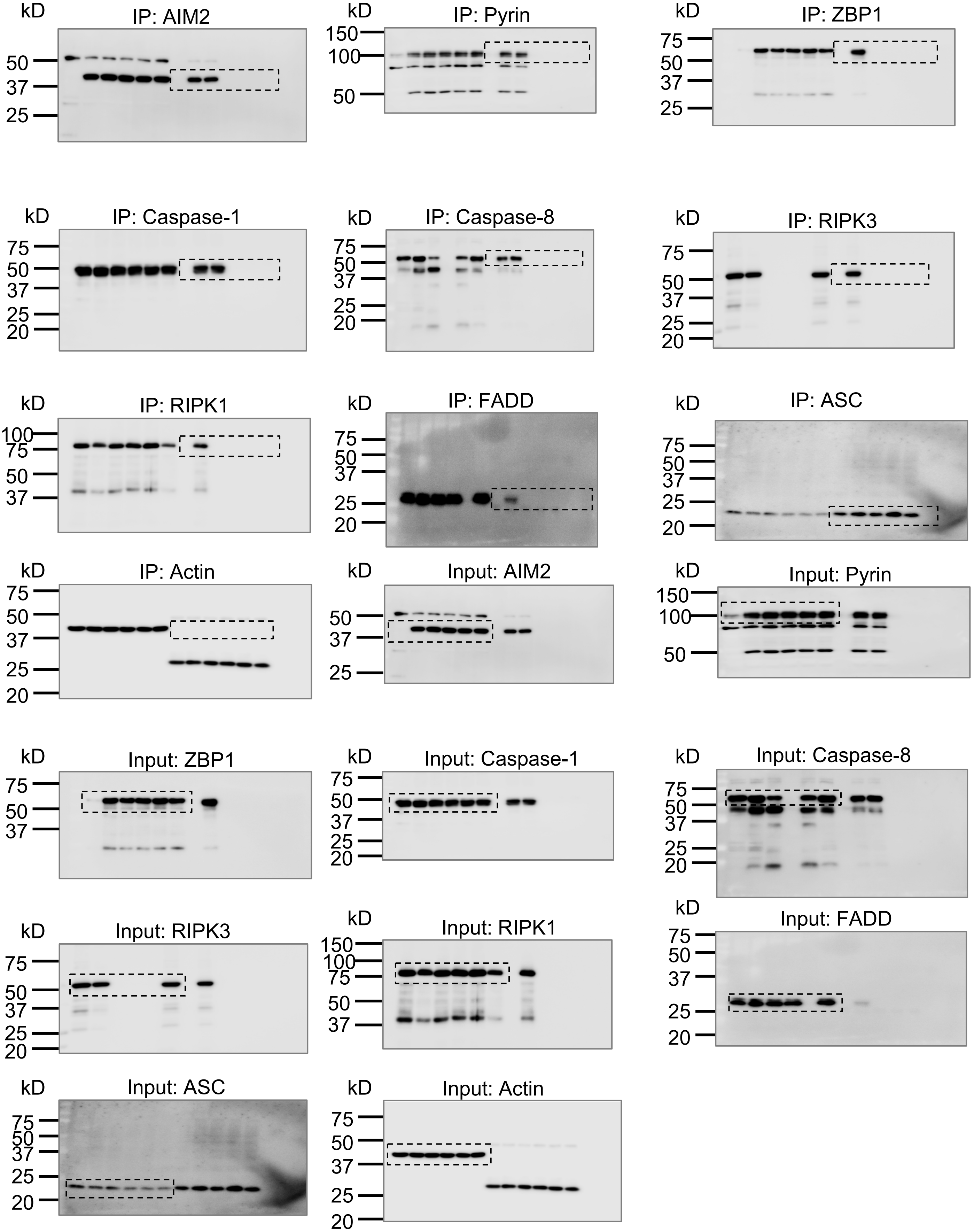

Extended Data Figure 8

d

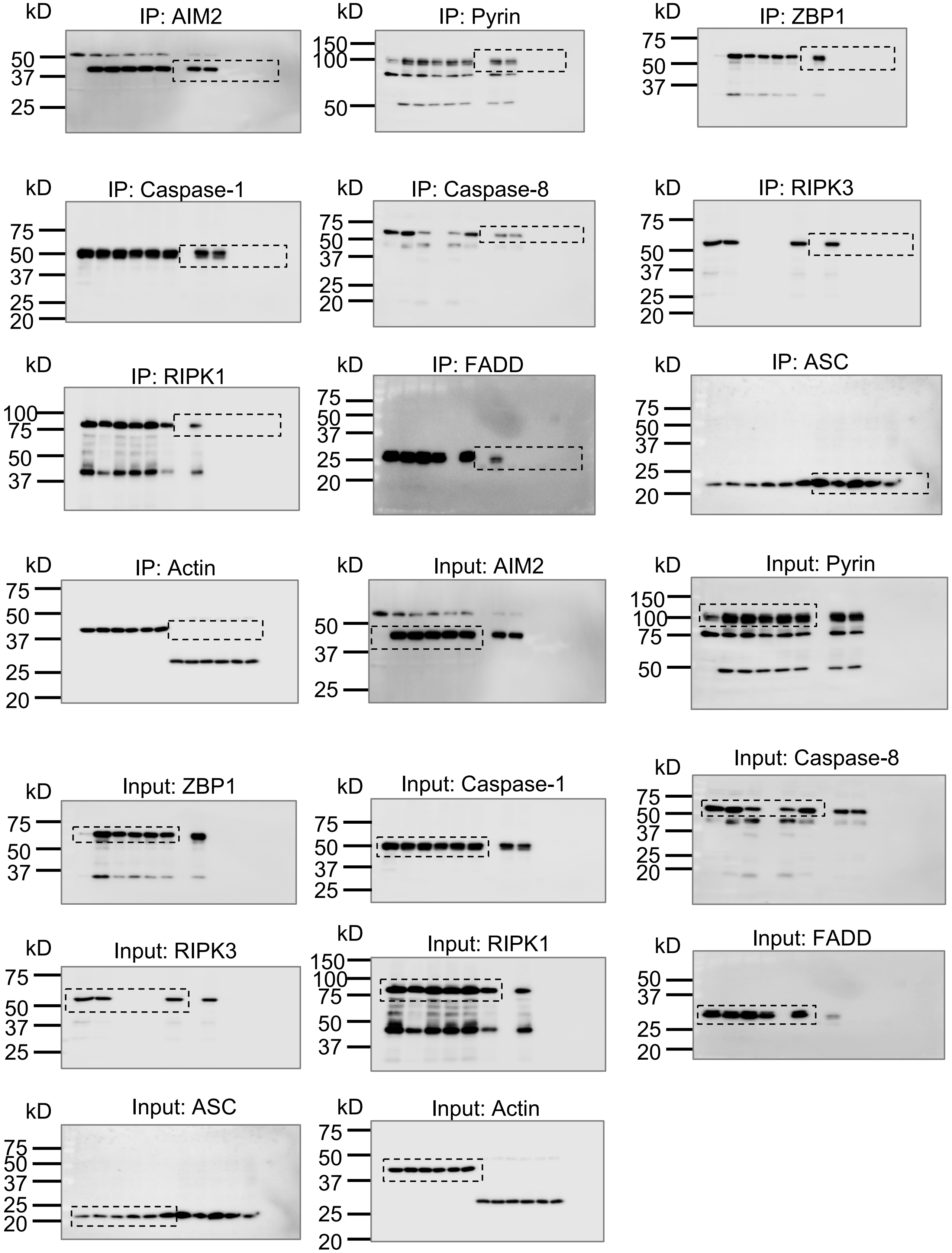

Extended Data Figure 8

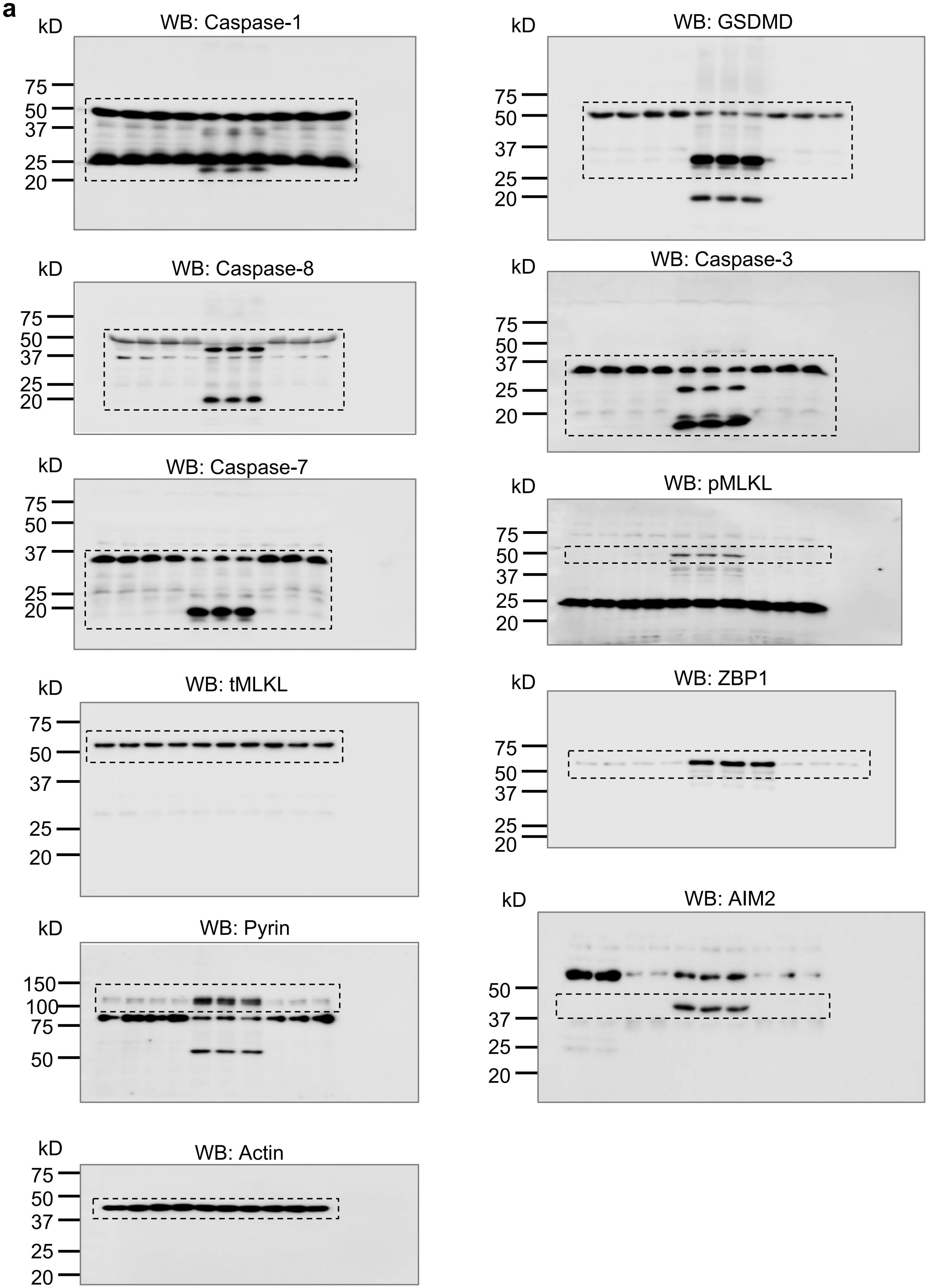

Extended Data Figure 10

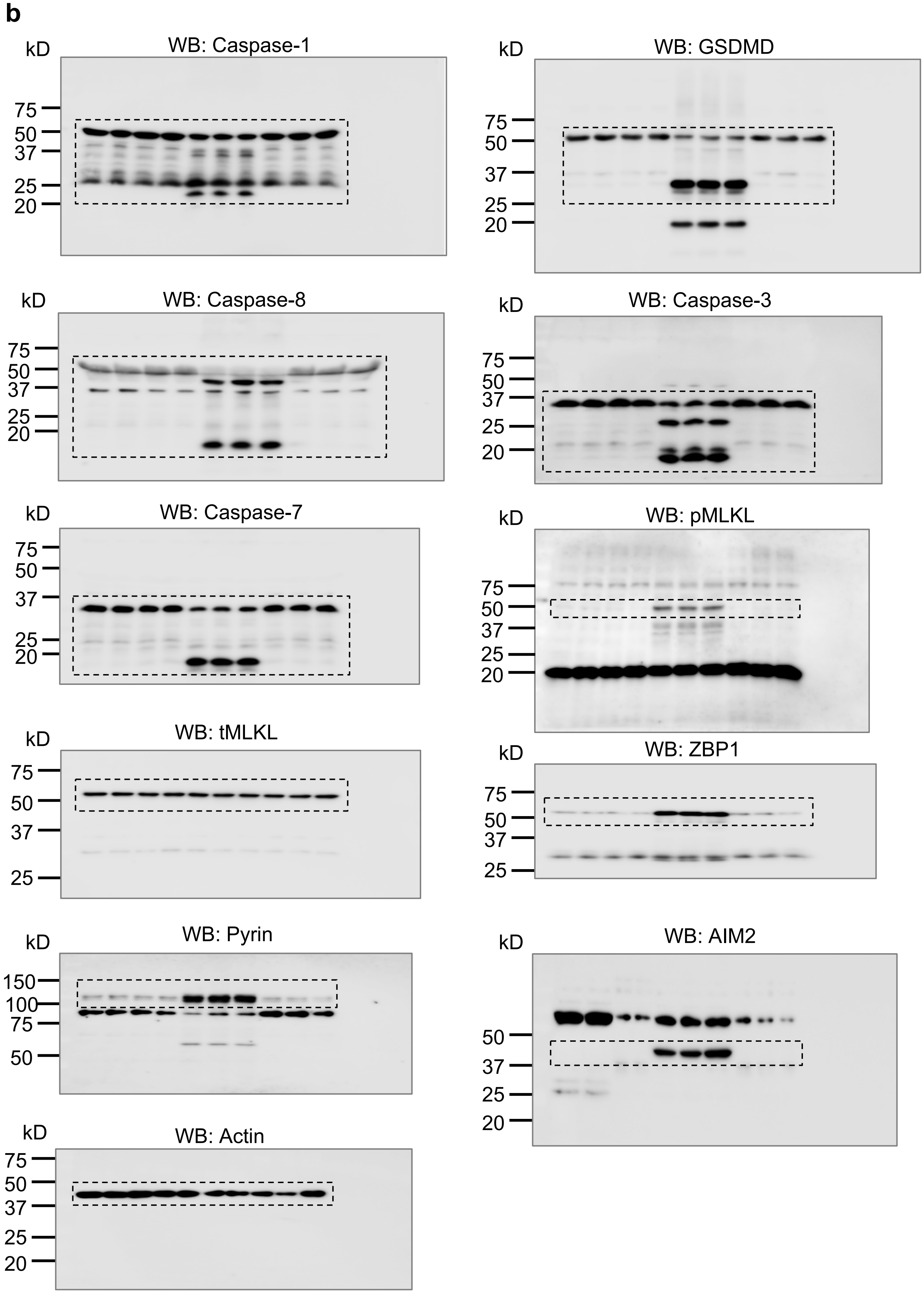

Extended Data Figure 10
