# Supplementary material for: AIM2 forms a complex with Pyrin and ZBP1 to drive PANoptosis and host defense
Source: Nature. Author manuscript; Available in PMC 2022 Sep 1. (PMC8603942; doi:10.1038/s41586-021-03875-8)
Supplement: Supplementary Table 1 [file NIHMS1739724-supplement-Supplementary_Table_1.pdf]

Supplementary Table 1: Exact *P* values.

**Figure 1b**	WT vs Aim2-/-	P value	< 0.0001
		Summary	****

**Figure 1c**	WT vs Aim2-/-	P value	< 0.0001
		Summary	****

**Figure 1e**	WT vs Aim2-/-	P value	< 0.0001
		Summary	****

**Figure 1g**	WT vs Nlrp3-/-	P value	0.3936
		Summary	ns

**Figure 1h**	WT vs Nlrp3-/-	P value	0.9997
		Summary	ns

**Figure 1j**	WT vs Nlrp3-/-	P value	0.9063
		Summary	ns

**Figure 1l**	WT vs Nlrc4-/-	P value	0.4283
		Summary	ns

**Figure 1m**	WT vs Nlrc4-/-	P value	0.7626
		Summary	ns

**Figure 1o**	WT vs Nlrc4-/-	P value	0.198
		Summary	ns

**Figure 1q**	WT vs Mefv-/-	P value	< 0.0001
		Summary	****

**Figure 1r**	WT vs Mefv-/-	P value	< 0.0001
		Summary	****

**Figure 1t**	WT vs Mefv-/-	P value	< 0.0001
		Summary	****

**Figure 1v**	WT vs Zbp1-/-	P value	< 0.0001
		Summary	****

**Figure 1w**	WT vs Zbp1-/-	P value	< 0.0001
		Summary	****

**Figure 1y**	WT vs Zbp1-/-	P value	< 0.0001
		Summary	****

Figure 2d		P Value	Summary
HSV1	WT vs Aim2-/-	< 0.0001	****
	WT vs Mefv-/-Zbp1-/-	< 0.0001	****
	Aim2-/- vs Mefv-/-Zbp1-/-	0.9976	ns
F. novicida	WT vs Aim2-/-	< 0.0001	****
	WT vs Mefv-/-Zbp1-/-	< 0.0001	****
	Aim2-/- vs Mefv-/-Zbp1-/-	0.9997	ns

Figure 2g		P Value	Summary
HSV1	WT vs Aim2-/-	< 0.0001	****
	WT vs Mefv-/-	< 0.0001	****
	WT vs Zbp1-/-	< 0.0001	****
	WT vs Mefv-/-Zbp1-/-	< 0.0001	****

Figure 2h		P Value	Summary
HSV1	WT vs Aim2-/-	< 0.0001	****
	WT vs Mefv-/-	< 0.0001	****
	WT vs Zbp1-/-	< 0.0001	****
	WT vs Mefv-/-Zbp1-/-	< 0.0001	****

Figure 2i		P Value	Summary
F. novicida	WT vs Aim2-/-	< 0.0001	****
	WT vs Mefv-/-	< 0.0001	****
	WT vs Zbp1-/-	< 0.0001	****
	WT vs Mefv-/-Zbp1-/-	< 0.0001	****

Figure 2j		P Value	Summary
F. novicida	WT vs Aim2-/-	< 0.0001	****
	WT vs Mefv-/-	< 0.0001	****
	WT vs Zbp1-/-	< 0.0001	****
	WT vs Mefv-/-Zbp1-/-	< 0.0001	****

Figure 4g		P Value	Summary
HSV1	Media vs HSV1	< 0.0001	****
	WT vs Asc-/-	< 0.0001	****
	WT vs Aim2-/-	< 0.0001	****
	WT vs Mefv-/-	< 0.0001	****
	WT vs Zbp1-/-	< 0.0001	****
	WT vs Mefv-/-Zbp1-/-	< 0.0001	****

Figure 4h	WT vs Aim2-/-	P value	0.0007
		Summary	***

Figure 4i		P Value	Summary

HSV1	WT vs Aim2-/-	< 0.0001	****
	WT vs Mefv-/-	0.0039	**
	WT vs Zbp1-/-	0.0004	***

**Extended Data Figure 1c**	WT vs Aim2-/-	P value	< 0.0001
		Summary	****

**Extended Data Figure 1f**	WT vs Nlrp3-/-	P value	0.432
		Summary	ns

**Extended Data Figure 1i**	WT vs Nlrc4-/-	P value	0.8526
		Summary	ns

**Extended Data Figure 1l**	WT vs Mefv-/-	P value	< 0.0001
		Summary	****

**Extended Data Figure 1o**	WT vs Zbp1-/-	P value	< 0.0001
		Summary	****

**Extended Data Figure 2c**		P Value	Summary
HSV1	WT vs Tlr3-/-	0.3205	ns
	WT vs Trif-/-	0.6823	ns

**Extended Data Figure 2f**		P Value	Summary
HSV1	WT vs Mda5-/-	0.9846	ns
	WT vs Mavs-/-	0.752	ns

**Extended Data Figure 2i**		P Value	Summary
HSV1	WT vs Nlrp12-/-	0.9409	ns
	WT vs Nlrp6-/-	0.8201	ns

**Extended Data Figure 2l**		P Value	Summary
F. novicida	WT vs Tlr3-/-	0.8943	ns
	WT vs Trif-/-	0.9686	ns

**Extended Data Figure 2o**		P Value	Summary
F. novicida	WT vs Mda5-/-	0.9627	ns
	WT vs Mavs-/-	0.9025	ns

**Extended Data Figure 2r**		P Value	Summary
F. novicida	WT vs Nlrp12-/-	>0.9999	ns
	WT vs Nlrp6-/-	0.4935	ns

Extended Data Figure 3b		P Value	Summary
HSV1	WT (HSV1) vs WT (HSV1+Col)	< 0.0001	****
	WT (HSV1+Col) vs Zbp1-/- (HSV1+Col)	< 0.0001	****
	Aim2 (HSV1+Col) vs Zbo1-/- (HSV1+Col)	0.3657	ns

Extended Data Figure 3d		P Value	Summary
F. novicida	WT (*F. novicida*) vs WT (F. novicida+Col)	< 0.0001	****
	WT (F. novicida+Col) vs Zbp1-/- (F. novicida+Col)	< 0.0001	****
	Aim2 (F. novicida+Col) vs Zbo1-/- (F. novicida+Col)	0.9996	ns

Extended Data Figure 3e		P Value	Summary
HSV1	WT (HSV1) vs WT (HSV1+Col)	< 0.0001	****
	WT (HSV1+Col) vs Zbp1-/- (HSV1+Col)	< 0.0001	****
	Aim2 (HSV1+Col) vs Zbo1-/- (HSV1+Col)	0.927	ns

Extended Data Figure 3f		P Value	Summary
HSV1	WT (HSV1) vs WT (HSV1+Col)	< 0.0001	****
	WT (HSV1+Col) vs Zbp1-/- (HSV1+Col)	< 0.0001	****
	Aim2 (HSV1+Col) vs Zbo1-/- (HSV1+Col)	0.9997	ns

Extended Data Figure 3g		P Value	Summary
F. novicida	WT (*F. novicida*) vs WT (F. novicida+Col)	< 0.0001	****
	WT (F. novicida+Col) vs Zbp1-/- (F. novicida+Col)	< 0.0001	****
	Aim2 (F. novicida+Col) vs Zbo1-/- (F. novicida+Col)	>0.9999	ns

Extended Data Figure 3h		P Value	Summary
F. novicida	WT (*F. novicida*) vs WT (F. novicida+Col)	< 0.0001	****
	WT (F. novicida+Col) vs Zbp1-/- (F. novicida+Col)	< 0.0001	****
	Aim2 (F. novicida+Col) vs Zbo1-/- (F. novicida+Col)	0.9998	ns

Extended Data Figure 4a		P Value	Summary
	WT vs Aim2-/-	< 0.0001	****

HSV1	WT vs Mefv-/-	0.0005	***
	WT vs Zbp1-/-	0.0006	***
	WT vs Mefv-/-Zbp1-/-	< 0.0001	****

**Extended Data Figure 4b**		P Value	Summary
F. novicida	WT vs Aim2-/-	< 0.0001	****
	WT vs Mefv-/-	0.01	*
	WT vs Zbp1-/-	0.0226	*
	WT vs Mefv-/-Zbp1-/-	< 0.0001	****

**Extended Data Figure 4d**		P Value	Summary
HSV1	WT vs Aim2 KD	< 0.0001	****
	WT vs Mefv KD	< 0.0001	****
	WT vs Zbp1 KD	< 0.0001	****
	WT vs Mefv KD Zbp1 KD	< 0.0001	****

**Extended Data Figure 4g**		P Value	Summary
HSV1	WT vs Aim2-/-	< 0.0001	****
	WT vs Mefv-/-	0.7113	ns
	WT vs Zbp1-/-	0.8425	ns
	WT vs Mefv-/-Zbp1-/-	0.9913	ns

**Extended Data Figure 5a**		P Value	Summary
WT	Media vs TcdB	< 0.0001	****
	Media vs HSV1	< 0.0001	****
Aim2-/-	Media vs TcdB	< 0.0001	****
	Media vs HSV1	0.7351	ns
Mefv-/-	Media vs TcdB	0.0005	***
	Media vs HSV1	0.0002	***
Zbp1-/-	Media vs TcdB	< 0.0001	****
	Media vs HSV1	< 0.0001	****

**Extended Data Figure 5d**		P Value	Summary
WT	Media vs Poly(dA:dT)	0.988	ns
	Media vs HSV1	0.0031	**

**Extended Data Figure 5h**		P Value	Summary
HSV1	WT vs Zbp1-/-	< 0.0001	****
	WT vs Zbp1$\Delta z\alpha 2/\Delta z\alpha 2$	< 0.0001	****
	WT vs Ripk3-/-	< 0.0001	****

**Extended Data Figure 5i**		P Value	Summary
F. novicida	WT vs Zbp1-/-	< 0.0001	****
	WT vs Zbp1$\Delta z\alpha 2/\Delta z\alpha 2$	< 0.0001	****
	WT vs Ripk3-/-	< 0.0001	****

Extended Data Figure 7a		P Value	Summary
HSV1	WT (Media) vs WT (HSV1)	< 0.0001	****
	WT vs Aim2-/-	< 0.0001	****
	WT vs Asc-/-	< 0.0001	****
	WT vs Casp1-/-	< 0.0001	****

Extended Data Figure 7b		P Value	Summary
F. novicida	WT (Media) vs WT (HSV1)	< 0.0001	****
	WT vs Aim2-/-	< 0.0001	****
	WT vs Asc-/-	< 0.0001	****
	WT vs Casp1-/-	< 0.0001	****

Extended Data Figure 7c		P Value	Summary
HSV1	WT (Media) vs WT (HSV1)	< 0.0001	****
	WT vs Aim2-/-	< 0.0001	****
	WT vs Asc-/-	< 0.0001	****
	WT vs Casp1-/-	< 0.0001	****

Extended Data Figure 7d		P Value	Summary
F. novicida	WT (Media) vs WT (HSV1)	< 0.0001	****
	WT vs Aim2-/-	< 0.0001	****
	WT vs Asc-/-	< 0.0001	****
	WT vs Casp1-/-	< 0.0001	****

Extended Data Figure 7e		P Value	Summary
HSV1	WT vs Aim2-/-	< 0.0001	****
	WT vs Asc-/-	< 0.0001	****
	WT vs Casp1-/-	< 0.0001	****

Extended Data Figure 7f		P Value	Summary
F. novicida	WT vs Aim2-/-	< 0.0001	****
	WT vs Asc-/-	< 0.0001	****
	WT vs Casp1-/-	< 0.0001	****

Extended Data Figure 9b		P Value	Summary
F. novicida	WT (Media) vs WT (*F. novicida*)	< 0.0001	****
	WT vs Asc-/-	< 0.0001	****
	WT vs Aim2-/-	< 0.0001	****
	WT vs Mefv-/-	< 0.0001	****
	WT vs Zbp1-/-	< 0.0001	****
	WT vs Mefv-/-Zbp1-/-	< 0.0001	****

Extended Data Figure 9d		P Value	Summary
	WT (Media) vs WT (HSV1)	< 0.0001	****

HSV1	WT vs Asc-/-	< 0.0001	****
	WT vs Ripk3-/-	< 0.0001	****
	WT vs Casp8-/-Ripk3-/-	< 0.0001	****

**Extended Data Figure 9f**		P Value	Summary
F. novicida	WT (Media) vs WT (F. novicida)	< 0.0001	****
	WT vs Asc-/-	< 0.0001	****
	WT vs Ripk3-/-	< 0.0001	****
	WT vs Casp8-/-Ripk3-/-	< 0.0001	****

**Extended Data Figure 10c**		P Value	Summary
HSV1	WT vs Aim2-/-	< 0.0001	****
	WT vs Mefv-/-	< 0.0001	****
	WT vs Zbp1-/-	< 0.0001	****
	WT vs Mefv-/-Zbp1-/-	< 0.0001	****

**Extended Data Figure 10d**		P Value	Summary
6 h	WT vs Aim2-/-	< 0.0001	****
	WT vs Mefv-/-	0.0218	*
	WT vs Zbp1-/-	0.0429	*
	WT vs Mefv-/-Zbp1-/-	< 0.0001	****
12 h	WT vs Aim2-/-	< 0.0001	****
	WT vs Mefv-/-	< 0.0001	****
	WT vs Zbp1-/-	< 0.0001	****
	WT vs Mefv-/-Zbp1-/-	< 0.0001	****

**Extended Data Figure 10e**		P Value	Summary
Lung	WT vs Aim2-/-	0.0008	***
Liver	WT vs Aim2-/-	0.0024	**
Spleen	WT vs Aim2-/-	0.0006	***

**Extended Data Figure 10f**		P Value	Summary
F. novicida	WT vs Aim2-/-	< 0.0001	****
	WT vs Mefv-/-	0.0064	**
	WT vs Zbp1-/-	0.0014	**
